# Dynamic functional networks in idiopathic normal pressure hydrocephalus: Alterations and reversibility by CSF tap test

**DOI:** 10.1002/hbm.25308

**Published:** 2020-12-09

**Authors:** Alessandra Griffa, Giulia Bommarito, Frédéric Assal, François R. Herrmann, Dimitri Van De Ville, Gilles Allali

**Affiliations:** ^1^ Department of Clinical Neurosciences, Division of Neurology Geneva University Hospitals and Faculty of Medicine, University of Geneva Geneva Switzerland; ^2^ Institute of Bioengineering Center of Neuroprosthetics, École Polytechnique Fédérale De Lausanne (EPFL) Geneva Switzerland; ^3^ Department of Rehabilitation and Geriatrics Geneva University Hospitals and University of Geneva Geneva Switzerland; ^4^ Department of Radiology and Medical Informatics University of Geneva Geneva Switzerland; ^5^ Department of Neurology, Division of Cognitive & Motor Aging Albert Einstein College of Medicine, Yeshiva University Bronx New York USA

**Keywords:** brain dynamics, brain plasticity, co‐activation pattern analysis, CSF tap test, default mode network, normal pressure hydrocephalus, resting state fMRI

## Abstract

Idiopathic Normal Pressure Hydrocephalus (iNPH)—the leading cause of reversible dementia in aging—is characterized by ventriculomegaly and gait, cognitive and urinary impairments. Despite its high prevalence estimated at 6% among the elderlies, iNPH remains underdiagnosed and undertreated due to the lack of iNPH‐specific diagnostic markers and limited understanding of pathophysiological mechanisms. INPH diagnosis is also complicated by the frequent occurrence of comorbidities, the most common one being Alzheimer's disease (AD). Here we investigate the resting‐state functional magnetic resonance imaging dynamics of 26 iNPH patients before and after a CSF tap test, and of 48 normal older adults. Alzheimer's pathology was evaluated by CSF biomarkers. We show that the interactions between the default mode, and the executive‐control, salience and attention networks are impaired in iNPH, explain gait and executive disturbances in patients, and are not driven by AD‐pathology. In particular, AD molecular biomarkers are associated with functional changes distinct from iNPH functional alterations. Finally, we demonstrate a partial normalization of brain dynamics 24 hr after a CSF tap test, indicating functional plasticity mechanisms. We conclude that functional changes involving the default mode cross‐network interactions reflect iNPH pathophysiological mechanisms and track treatment response, possibly contributing to iNPH differential diagnosis and better clinical management.

## Graphical abstract

Idiopathic normal pressure hydrocephalus (iNPH), the leading cause of reversible dementia in ageing, is a prevalent neurological disorder characterised by gait, urinary, and cognitive impairments with ventriculomegaly. We show that iNPH patients have changes of brain functional dynamics which explain gait and executive deficits, are partially reverted by a CSF tap test, and are not driven by Alzheimer's disease or cerebrovascular comorbidities. Our results shed new light on the neurobiological substrates of iNPH and functional plasticity mechanisms following an invasive intervention, with possible implications for iNPH differential diagnosis and better clinical management.

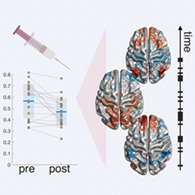

## 1 INTRODUCTION

Idiopathic normal pressure hydrocephalus (iNPH) is characterized by gait, cognitive and urinary impairments with ventriculomegaly at brain imaging (Gallia, Rigamonti, & Williams, 2006; Relkin, Marmarou, Klinge, Bergsneider, & Black, 2005; Williams & Malm, 2016). Symptoms can be partially reverted by CSF flow diversion with invasive shunt neurosurgery, making iNPH the leading cause of reversible dementia (Andrén et al., 2020; Halperin, Kurlan, Schwalb, & Cusimano, 2015; Todisco et al., 2020; Wallenstein & McKhann, 2010). The prevalence of iNPH is estimated at 6% in adults older than 80 years (Jaraj et al., 2014). However, only 8% of iNPH patients in the United States receive disease‐specific treatment (Halperin et al., 2015). iNPH is largely underdiagnosed and undertreated because of lack of accurate diagnostic and prognostic quantitative biomarkers, frequent presence of comorbidities, and limited understanding of the etiological and pathophysiological processes underlying the disorder (Lu et al., 2020; Martín‐Láez, Caballero‐Arzapalo, López‐Menéndez, Arango‐Lasprilla, & Vázquez‐Barquero, 2015; Oliveira et al., 2019).

The diagnosis of iNPH is challenging because symptoms and radiological features can be confused with alternate neurological conditions; for example, Parkinson's disease or Alzheimer's disease (AD). Gait disturbance, the key symptom of iNPH, is not specific (Morel, Armand, Assal, & Allali, 2019) and frequently found in other parkinsonian syndromes, while cognitive impairments (poor executive function and memory) and brain tissues compression caused by ventriculomegaly can be misinterpreted by AD‐related cognitive decline and atrophy (Malm et al., 2013). To overcome these bottlenecks, efforts have been devoted to identifying iNPH‐specific neuroimaging markers based on brain‐circuits characterization (Griffa, Van De Ville, Herrmann, & Allali, 2020; Hoza, Vlasák, Hořínek, Sameš, & Alfieri, 2015; Siasios et al., 2016; Tarnaris, Kitchen, & Watkins, 2009). Diffusion‐weighted magnetic resonance imaging studies highlighted periventricular, frontoparietal, and cortico‐subcortical white matter (WM) microstructural alterations compatible with the mechanical and ischemic damage associated with iNPH (Kamiya et al., 2017; Siasios et al., 2016) and able to discriminate iNPH from other neurodegenerative disorders (Hattori et al., 2011; Hattori, Sato, Aoki, Yuasa, & Mizusawa, 2012; Kim et al., 2011). A subset of studies also demonstrated WM changes after shunt surgery or CSF tap test, indicating a possible short‐term structural response to CSF drainage (Jurcoane et al., 2014; Kamiya et al., 2017; Kanno et al., 2017; Saito et al., 2020). However, the associations of WM features with iNPH symptoms, or with clinical improvement after treatment, are not reproducible across studies. Little is known about brain functional alterations in iNPH. As we have recently reviewed, few electroencephalography and functional magnetic resonance imaging (fMRI) studies report alterations of brain dynamics across multiple brain regions including frontal, motor, occipital, temporal, and cingular areas, but findings remain poorly consistent (Griffa et al., 2020).

A better characterization of brain functional alterations in iNPH is desirable for several reasons. First, the comprehension of the functional dynamics associated with iNPH symptoms and response to treatment, could shed new light on the pathophysiological mechanisms, and contribute to improve diagnostic guidelines and clinical management. Functional connectivity features have higher behavior predictive capacity than brain structural features (Amico & Goñi, 2018; Finn et al., 2015; Lin, Baete, Wang, & Boada, 2020) and are, therefore, likely to have better correlation with symptoms. In particular, including both spatial and temporal (besides static connectivity) features of the occurring functional reorganizations allows to achieve a better classification of neurodegenerative disorders (de Vos et al., 2018; Liégeois et al., 2019; Preti, Bolton, & Van De Ville, 2017). Moreover, functional features may be more sensitive to short‐term plasticity mechanisms occurring after CSF drainage. Lastly, the lack of specificity of symptoms and the high prevalence of comorbidities observed in iNPH suggests, on one side, the presence of dimensional symptom‐level functional abnormalities (possibly shared across disorders), and, on the other side, the co‐existence of multiple pathological pathways leading to possibly distinct functional alterations. The analysis of CSF molecular markers, including the core AD biomarkers amyloid‐𝛽 42, phosphorylated tau and total tau, has proved effective for the differential diagnosis of iNPH from cognitive, movement and cerebrovascular mimic disorders (Jeppsson et al., 2019; Manniche, Hejl, Hasselbalch, & Simonsen, 2019). However, it is currently unknown how comorbid pathologies, and, in particular, amyloid‐ and tau‐pathways, contribute to the brain functional alterations observed in iNPH. In sum, functional networks in iNPH might not only contribute to a better understanding of iNPH mechanisms but also more importantly help the clinicians to predict the best responders to shunt surgery.

Here we tackle the open questions of iNPH disorder—the need for quantitative neuroimaging markers, the comprehension of brain changes following treatment, and the influence of AD pathology on its pathophysiological mechanisms—from the perspective of brain functional circuits probed with resting‐state fMRI. To this end, iNPH patients are enrolled into a well‐established 2‐day protocol with gait, neuropsychological and MRI assessments before and after a CSF tap test (Allali et al., 2017). The aims of the study are (a) to identify functional circuits related to iNPH disorder and investigate their spatial and temporal characteristics over resting‐state recordings; (b) to explore the relationship between iNPH functional neuroimaging features and clinical dimensions; (c) to assess whether iNPH functional alterations are reverted by CSF tap test; and (d) to understand whether iNPH functional alterations are driven by AD pathways, the most common neurological comorbidity of iNPH. By jointly tackling these open questions, we aim to provide a comprehensive and informative picture of the resting‐state functional characteristics of iNPH.

## 2 MATERIALS AND METHODS

### 2.1 Participants

Thirty‐one iNPH patients and 48 healthy controls (HCs) were recruited at Geneva University Hospital, Department of Neurology, between March 2017 and February 2020 according to a previously described protocol (Allali et al., 2017). Briefly, inclusion criteria for patients were a diagnosis of possible or probable iNPH, ability to walk without assistance, and no contraindication for MRI. The diagnosis of iNPH was performed at a consensus case conference involving behavioral neurologists and neuropsychologists, and based on international consensus guidelines (Relkin et al., 2005). Exclusion criteria were the presence of an acute medical illness in the past 3 months, orthopedic disorders interfering with gait, and a diagnosis of secondary normal pressure hydrocephalus. Five patients were excluded because of corrupted MRI data (*N* = 1) or excessive movement during the MRI scan (*N* = 4). Eventually, the study included a total of 26 iNPH patients (mean age 79.7 ± 6.3 years, 12 women) and 48 HCs (mean age 74.9 ± 5.5 years, 36 women) (Table 1). The study was approved by the ethical committee of Geneva University Hospitals (protocol NAC11‐125) and all subjects provided informed consent according to the Declaration of Helsinki.

**TABLE 1:** Study participants

	HC	iNPH (pre‐CSF tap test)	iNPH (post‐CSF tap test) test)	*p*‐value HC/iNPH	*p*‐value pre‐/post‐ CSF tap test
	*N* = 48	*N* = 26	*N* = 21		
Age (years)	74.5 (5.5)	78.3 (7.7)	–	.061	–
Gender, female (*n*)	32	13	–	.042 *	–
Handedness, right‐handed (*n*)	38	29	–	.10	–
Education level, I/II/III (*n*)	4/13/26	18/6/5	–	6.5e−07 ***	–
A𝛽_1–42_ (ng/L)	–	699.8 (265.4)	–	–	–
Total tau (ng/L)	–	238.1 (115.5)	–	–	–
pTau (ng/L)	–	43.4 (15.7)	–	–	–
WM hypointensities (ICV %)	0.27% (0.34%)	0.92% (0.79%)	–	5.7e−05 ***	–
fMRI average FD (mm)	0.22 (0.07)	0.26 (0.09)	0.26 (0.10)	.11	.37
fMRI motion‐corrupted volumes (*n*)	5 (10)	15 (25)	18 (31)	.015 *	.15
**Gait**					
Walking speed—mean (m/s)	1.22 (0.14)	0.71 (0.26)	0.79 (0.31)	3.8e−14 ***	.013 *
Stride time—mean (s)	1.08 (0.10)	1.25 (0.18)	1.19 (0.18)	6.0e−05 ***	.042 *
Stride length—mean (m)	1.31 (0.10)	0.87 (0.26)	0.91 (0.30)	3.1e−15 ***	.007 *
Step width—mean (m)	0.07 (0.03)	0.10 (0.03)	0.11 (0.04)	.00075 **	.44
**Global cognitive functioning**					
MMSE [0–30]	27.1 (2.0)	23.7 (4.4)	–	.029 *	–
**Memory**					
FCSRT immediate free recall [0–48]	25.9 (7.3)	14.9 (7.8)	–	8.4e−05 ***	–
FCSRT delayed free recall [0–16]	10.2 (2.3)	5.6 (3.4)	–	.00035 **	–
FCSRT immediate total recall [0–48]	43.5 (5.2)	37.2 (8.2)	–	.015 *	–
FCSRT delayed total recall [0–16]	14.7 (1.8)	12.2 (4.1)	–	.0076 *	–
**Executive functions**					
Color Trail test—Part A (s)	58.7 (15.4)	123.8 (63.4)	116.2 (66.3)	2.7e−05 ***	.94
Color Trail test—Part B (s)	122.4 (45.1)	228.9 (85.1)	219.4 (100.6)	6.5e−06 ***	.15
Color Trail test—Index (%)	1.15 (0.71)	1.27 (1.05)	1.13 (0.78)	.89	.31
Verbal fluency–Categorical (*n*)	19.5 (4.8)	12.0 (3.9)	11.6 (3.9)	3.0e−05 ***	.69
Verbal fluency–Phonemic (*n*)	19.0 (6.9)	10.0 (7.0)	12.3 (5.6)	.00046 **	.29
**Attention**					
WAIS‐III symbol digit modalities (*n*)	54.1 (16.0)	29.2 (12.4)	34.6 (11.9)	2.0e−05 ***	.015 *
WAIS‐III forward digit span (*n*)	6.0 (1.2)	5.2 (0.9)	5.4 (1.0)	.069	.99
WAIS‐III backward digit span (*n*)	4.1 (1.2)	3.7 (0.9)	3.5 (1.1)	.56	.23
WMS‐III forward visual span (*n*)	5.0 (1.1)	4.2 (1.3)	4.7 (1.3)	.38	.21
WMS‐III backward visual span (*n*)	4.3 (0.7)	3.4 (0.9)	3.6 (0.9)	.021 *	.18

*Note*: Group differences between iNPH and HC groups were assessed with Student's *t* test for continuous variables and chi‐square test for categorical variables. Group differences for gait and cognitive scores were assessed with ANCOVA, including age, gender, and education level as covariates. Differences between pre‐ and post‐CSF tap test data in iNPH patients were assessed with paired Student's *t* test. Gait and cognitive data were missing for some subjects, who were discarded from related group‐comparisons: gait assessment (*n* = 3 pre‐/*n* = 2 post‐CSF tap test); MMSE (*n* = 1); FCSRT, all scores (*n* = 2); Color Trail test, Part B and Index (*n* = 4 pre‐/*n* = 3 post‐CSF tap test); WAIS‐III all scores (*n* = 1 pre‐/*n* = 1 post‐CSF tap test). Group average (standard deviation) values are reported when appropriate. Units of measure are reported in round brackets (*n* = number); score intervals are reported in square brackets.

Abbreviations: FCSRT, French version of the free and cued selective reminding test; FD, framewise displacement; ICV, intracranial volume; MMSE, mini‐mental state examination; WAIS‐III, Wechsler Adult Intelligence Scale; WM, white matter.

* *p* < .05,

** *p* < .001,

*** *p* < .0001.

### 2.2 Experimental protocol with CSF tap test

iNPH patients underwent clinical and MRI assessments on two consecutive days. The first‐day assessment included a quantitative gait analysis, a neuropsychological test battery, an MRI acquisition and, finally, the CSF tap test. The tap test consisted in the removal of 40 ml of CSF with a 20‐gauge spinal needle with the patient lying in lateral supine position at the same time of the day. During the second day, patients underwent again the gait assessment, neuropsychological tests and an MRI acquisition. Of the 26 iNPH patients, 5 were excluded from post‐CSF tap test analyses because of corrupted (*N* = 1) or missing (*N* = 4) second‐day MRI data. HCs went through the same gait, neuropsychological and MRI assessments.

### 2.3 Assessment of gait and cognition

Quantitative spatiotemporal gait assessment was performed in a kinesiology laboratory. Subjects were equipped with reflective markers placed on the heels and asked to walk at their self‐selected speed on a 10‐m walkway. Reflective marker trajectories were recorded with a 12‐camera optoelectronic system to compute average gait parameters including walking speed, stride time, stride length, and step width (Allali et al., 2013; Armand et al., 2011). A standardized neuropsychological assessment was administered to patients and controls and included evaluation of executive functions (Color Trail test (D'Elia, Satz, Lyons Uchiyama, & White, 1996), categorical and phonemic verbal fluency (Cardebat, Doyon, Goulet, & Joanette, 1990)), attention (Wechsler Adult Intelligence Scale symbol digit test, digit span, and Memory Scale spatial span (Drozdick, Wahlstrom, Zhu, & Weiss, 2012)), and memory (French version of the Free and Cued Selective Reminding Test (Van Der Linden, Coyette, & Poitrenaud, 2004)). Global cognitive functioning was assessed with the Mini‐Mental State Examination (Folstein, Folstein, & McHugh, 1975). After the CSF tap test, patients were re‐assessed for executive functions, attention, and gait.

### 2.4 MRI data acquisition and processing

#### 2.4.1 MRI protocol

Subjects underwent MRI session(s) including structural and a resting‐state functional scans on a Siemens MAGNETOM Prisma^fit^ 3 T‐scanner equipped with a 64‐channel head coil. The structural scan consisted of a T1‐weighted magnetization‐prepared rapid acquisition gradient echo (MPRAGE) sequence with 0.8 mm isotropic voxel size (Table S1). The resting‐state functional scan consisted of a multiband accelerated echo planar imaging sequence sensitive to blood oxygen‐level‐dependent contrast with 2.5 mm isotropic voxel size, 64 slices, repetition time 1,057 ms, acquisition time 10 min 43 s (600 volumes were acquired for each scan) (Table S2). Moreover, a double‐echo gradient echo field map was acquired to estimate field inhomogeneities inside the scanner (Hutton et al., 2002) (Table S3). During the fMRI acquisition, subjects were instructed to keep their eyes closed without focusing on any specific task and without falling asleep.

#### 2.4.2 Processing of structural MRI data

MRI data were processed according to state‐of‐the‐art pipelines using *fMRIPrep* 1.5.0 (Esteban et al., 2019), *ANTs* 2.2.0 (Avants, Epstein, Grossman, & Gee, 2008), *FreeSurfer* 6.0.0 (Fischl, 2012), *FSL 6.0* (Jenkinson, Bannister, Brady, & Smith, 2002) and custom *Python* code. Briefly, individual T1‐weighted volumes were corrected for intensity nonuniformity, skull‐stripped and spatially normalized to MNI standard space (ICBM152 nonlinear asymmetrical template, v2009c (Fonov, Evans, McKinstry, Almli, & Collins, 2009)). Segmentation of CSF, gray matter, WM and WM hypointensities was performed with *FreeSurfer* 6.0.0 (Fischl et al., 2004). Individual cortical volumes were parcellated into 100 regions of interest grouped into 17 resting‐state networks (RSNs) (Schaefer et al., 2018; Yeo et al., 2011) (Figure S1). Spatial normalization and brain segmentation outcomes were visually inspected: one iNPH patient was excluded because of segmentation failure.

#### 2.4.3 Processing of resting‐state functional MRI data

FMRI volumes were non‐linearly corrected for susceptibility distortions estimated from the field map (Esteban et al., 2019), temporally realigned to correct for head motion (Jenkinson et al., 2002), and slice‐timing corrected (Cox & Hyde, 1997) using a unique composite geometric transformation. The first six volumes were discarded to allow signal stabilization and the remaining volumes were warped to the MNI space (Greve & Fischl, 2009; Jenkinson & Smith, 2001). Single‐voxel time series were corrected for nuisance contributions by regressing out the average WM and CSF signals, the six motion signals (three translations and three rotations) and low‐frequency components estimated with the discrete cosine transform (Lund, Madsen, Sidaros, Luo, & Nichols, 2006; Power et al., 2014). In particular, the three nonzero lowest frequency basis vectors of the discrete cosine transform were removed, corresponding to oscillations at 0.0008, 0.0017, and 0.0025 Hz. Finally, a spatial Gaussian smoothing of 6 mm full‐width‐at‐half‐maximum and a temporal band‐pass filter with 0.01–0.15 Hz band limits were applied. Analyses were repeated while including the global signal (average of fMRI time series over brain voxels) as additional regressor (Murphy & Fox, 2017). Registrations and susceptibility distortion corrections were visually inspected.

#### 2.4.4 Global signal

The fMRI global signal has been associated with both artifacts (respiratory and heartbeat oscillations, hardware fluctuations) (Chang & Glover, 2009; Fox, Zhang, Snyder, & Raichle, 2009; Murphy, Birn, Handwerker, Jones, & Bandettini, 2009) and neural activity (Schölvinck, Maier, Ye, Duyn, & Leopold, 2010). Moreover, recent studies associate the global signal with behavioral traits (Li et al., 2019b), pathological conditions (Scalabrini et al., 2020; Wang et al., 2019; Yang et al., 2014), and more generally to the anticorrelation patterns observed between the default mode and task‐positive network. The interpretation of the global signal is therefore debated (Murphy & Fox, 2017). For this reason, we adopted two strategies to assess the influence of the global signal on our data. First, we repeated all the analyses while including the global signals as an additional regressor (besides the WM, CSF, motion, and low‐frequency components) in the preprocessing pipeline. Second, we investigated whether the representation of the global signal in individual subjects is modulated by iNPH pathology (the computation of the global signals representation maps is described below).

#### 2.4.5 Head motion

Head motion is a severe confounding factor of functional connectivity analyses and needs to be carefully considered, particularly when investigating elderly populations prone to high levels of motion (Gratton et al., 2020). The framewise displacement (FD) was used as motion indicator (Power, Barnes, Snyder, Schlaggar, & Petersen, 2012) and fMRI volumes with FD > 0.7 mm, together with their precedent and two following volumes, were discarded from the computation of functional measures. Moreover, 4 iNPH patients with less than 4 min of uncorrupted fMRI recoding after FD censoring were excluded. The final iNPH (*N* = 26) and HC (*N* = 48) groups, and the iNPH pre‐ and post‐CSF tap test (*N* = 21) groups did not differ in terms of average FD, although a larger proportion of fMRI volumes was discarded in iNPH patients compared with controls (Table 1). All statistical analyses were replicated including the number of excluded fMRI volumes as covariate.

### 2.5 Analysis of resting‐state functional dynamics

To identify the brain circuits affected in iNPH and characterize their spatial and temporal characteristics during resting‐state, we developed a three‐step methodological approach. First, we performed a whole‐brain analysis of within‐ and between‐RSNs functional connectivity. Results identified the default mode (DMN) as the central network vulnerable to iNPH. Second, we investigated the spatial characteristics of the average co‐activation map of the precuneus, the main DMN hub (Raichle, 2015), and the spatial representation of the global signal (Murphy & Fox, 2017). Third, we clustered in time the precuneus co‐activations to unravel the dynamic interactions between this hub and other DMN regions, as well as other functional circuits. The latter analysis delivers interpretable statistics that can be compared before and after the CSF tap test and related to AD pathways and clinical dimensions.

#### 2.5.1 Step 1: Assessment of resting‐state network connectivity

**FIGURE 1:**
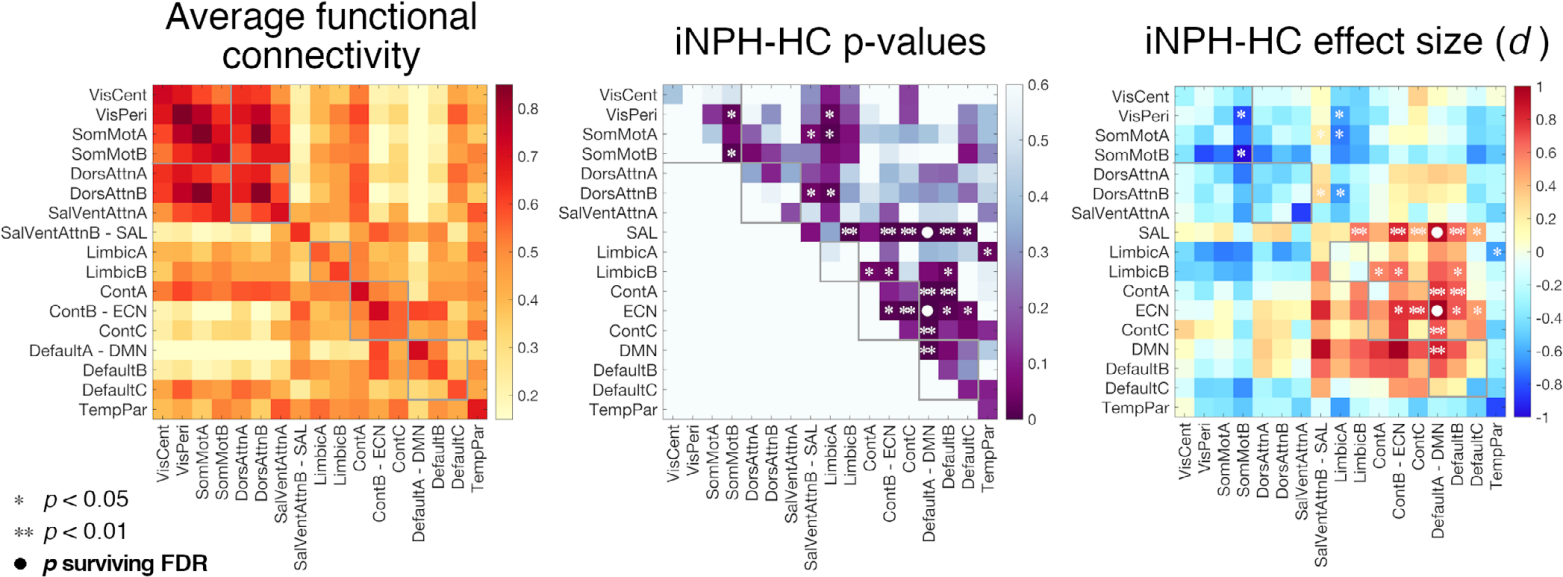
Resting‐state network connectivity in iNPH patients and healthy controls. From left to right: average within‐ and between‐RSN functional connectivity values across subjects; *p*‐values (ANCOVA) and Cohen's *d* effect sizes for the iNPH (pre‐CSF tap test assessment)‐HC group‐comparisons. RSNs labels follow the convention of (Schaefer et al., 2018; Yeo et al., 2011). Gray lines delineate the visual‐somatomotor, attention, limbic, control and default mode brain systems. *p < 0.05; **p < .01; ● *p* surviving FDR‐correction (*q* < 0.05)

Hundred cortical regions of interest were associated with fMRI time series by averaging the pre‐processed fMRI data over the voxels belonging to each region. The functional connectivity between region pairs was quantified with the Pearson's correlation coefficient between the time series and averaged over the regions belonging to the different RSNs to deliver individual 17 × 17 functional connectivity matrices (Figure 1a). Analyses were replicated with an alternative parcellation including 400 regions (Schaefer et al., 2018) (Figure S2).

#### 2.5.2 Step 2: Precuneus functional co‐activation maps and global signal representation

**FIGURE 2:**
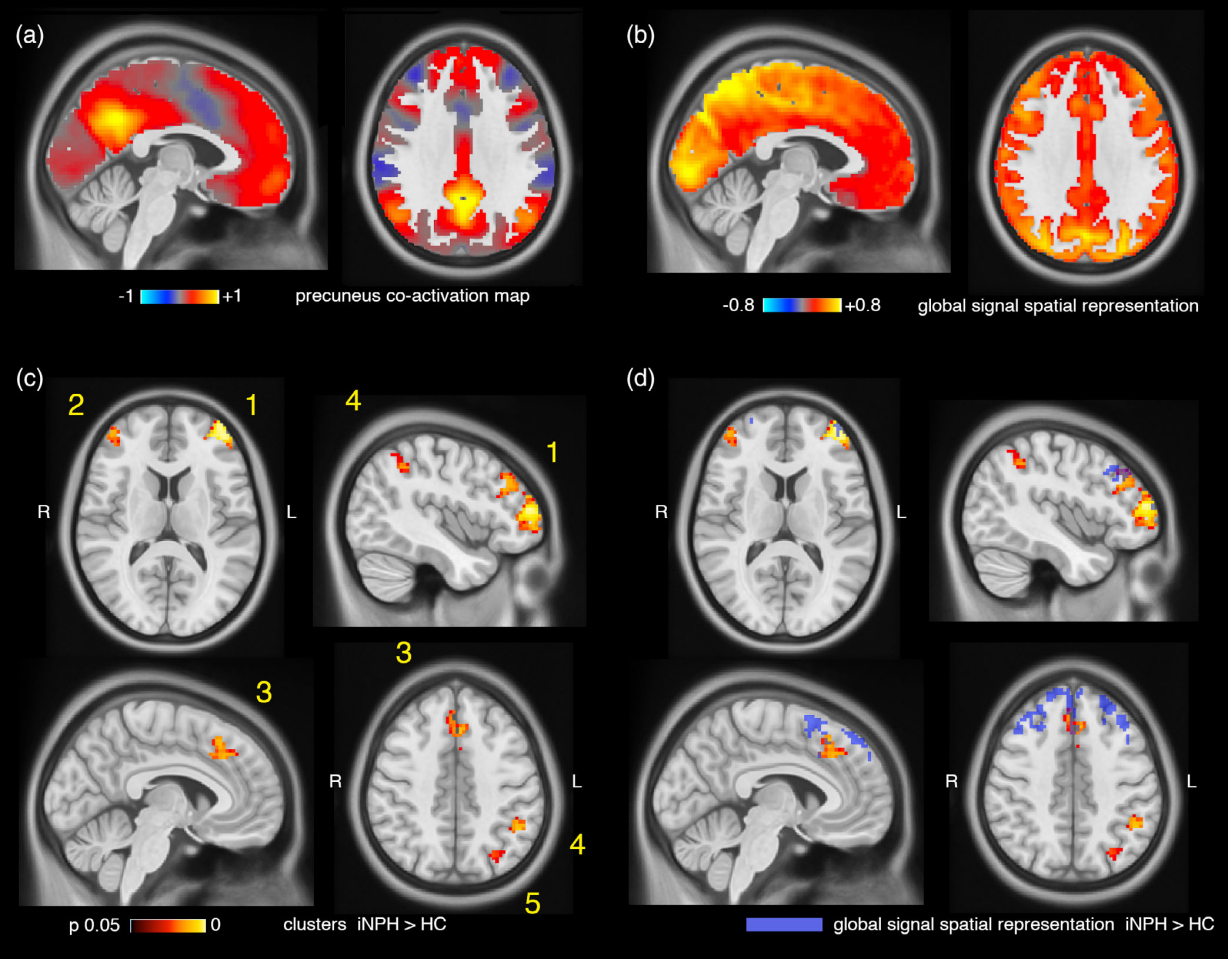
Cortical clusters of increased co‐activation with the precuneus. (a) Group‐average precuneus co‐activation map from iNPH patients and healthy controls. The color scale represents average fMRI signal values over fMRI volumes corresponding to precuneus activations. (b) Group‐average global signal representation map from iNPH patients and healthy controls. The color scale represents average fMRI signal values over fMRI volumes corresponding to time point of high global signal (> 1 *SD*). (c) Cortical clusters of significantly increased co‐activation with the precuneus in iNPH compared with HCs. The color scale represents voxel‐level multiple‐comparison corrected *p*‐values. (d) Cortical clusters of significantly increased co‐activation with the precuneus superimposed with clusters of increased global signal representation (in blue) in iNPH compared with HCs

Two spheres of radius 6.25 mm were placed in the centroids of the left and right medial posterior hubs of the DMN (Schaefer et al., 2018; Yeo et al., 2011), corresponding to the precunei/posterior cingulate cortices (MNI coordinates −5.7,−54.3,34.1; 7.5,−53.7,31.2; Figure S3). The seed time series were extracted from the pre‐processed fMRI data, z‐scored and thresholded at 1 *SD* to identify the time points corresponding to precuneus high‐amplitude events (or activations) in the blood oxygenation level‐dependent signal (Liu & Duyn, 2013; Tagliazucchi, Balenzuela, Fraiman, & Chialvo, 2012). For each subject, the fMRI volumes corresponding to precuneus activations were then averaged in time to obtain individual precuneus co‐activation maps (Figure 2a), which largely summarize seed‐based functional connectivity patterns (Perri et al., 2018; Tagliazucchi, Siniatchkin, Laufs, & Chialvo, 2016). Individual spatial maps of global signal representation were obtained with a similar procedure but using the global signal extracted from pre‐processed fMRI data as seed time course. The global signal was z‐scored and thresholded at one *SD*, and the fMRI volumes corresponding to time points with above‐threshold global signal were averaged in time to obtain individual global signal representation maps (Figure 2b).

#### 2.5.3 Step 3: Temporal characterization of DMN dynamics

The fMRI volumes selected by precuneus activations were clustered into three states using the *k*‐means algorithm. The z‐scored centroids of the clusters represent distinct precuneus co‐activation patterns (CAPs) with positive and negative cortical contributions (Liu & Duyn, 2013; Liu, Zhang, Chang, & Duyn, 2018). The relative temporal occurrence (defined as the number of fMRI volumes classified in one CAP‐cluster normalized by the number of volumes corresponding to precuneus activations), average duration (the average number of consecutive volumes classified in one CAP‐cluster) and frequency (the number of disjoint volume‐sets classified in one CAP‐cluster normalized by the recording time) of the three CAPs were computed for each subject. The choice of the number of clusters (*k* = 3) was based on a consensus clustering assessment from data bootstrapping (Bolton et al., 2019; Monti, Tamayo, Mesirov, & Golub, 2003) and ensuring a minimum cluster expression across subjects (Figure S4). Analyses were replicated with an alternative number of clusters (*k* = 4).

### 2.6 AD and white matter pathologies

AD‐related molecular pathways were assessed in iNPH patients based on A𝛽_1–42_ (the 42 amino acid form of 𝛽‐amyloid), phosphorylated tau (pTau) and total tau levels in the CSF (Blennow, Vanmechelen, & Hampel, 2001). Briefly, CSF samples of 10 ml were centrifuged at 4°C for 10 min at 2000*g* within 4 hr from lumbar puncture, aliquoted into 0.5‐ml polypropylene tubes and stored at −80°C until analysis (Allali, Laidet, Armand, & Assal, 2018b). A𝛽_1–42_, pTau and total Tau were measured in duplicate using a double‐sandwich enzyme‐linked immunosorbent assay (INNOTEST, Fujirebio) (Table 1). Total Tau and pTau were strongly correlated in our sample (Pearson's correlation coefficient *r*[24] = .94, *p* < 10^−11^) and further analyses focused on pTau and A𝛽_1–42_ only. AD pathology was assessed according to consolidated cut‐off levels: amyloid‐positivity was defined as A𝛽_1–42_ < 450 ng/L for subjects younger than 70 years and A𝛽_1–42_ < 500 ng/L for subject older than 70 years (Sjögren et al., 2001); tau‐positivity was defined as pTau>61 ng/L (Tariciotti et al., 2018).

WM hypointensity volumes normalized by individual intracranial volumes were used as indicator of WM degeneration and possible cerebrovascular WM‐disease comorbidity (Table 1). WM hypointensities identified from T1‐weighted sequence are highly correlated to manual and semi‐manual measurements from T2 or FLAIR sequences and have similar correlation with age and CSF biomarkers in healthy elderly (Bagnato et al., 2003; Wei et al., 2019).

### 2.7 Statistical analyses

Statistical differences between iNPH and control groups were assessed with analysis of covariance (ANCOVA) including age, gender, and education level as covariates. Statistical differences between pre‐ and post‐CSF tap test assessments were evaluated with paired *t* test. Effect sizes were quantified with the Cohen's *d*. Pair‐wise relationships were assessed with the Pearson's correlation coefficient (*r*). Multiple comparison correction was applied when necessary using the Benjamini‐Hochberg procedure to control the false discovery rate (FDR) at *q* < 0.05 (Benjamini & Hochberg, 1995; Benjamini & Yekutieli, 2001).

#### 2.7.1 Voxel‐wise precuneus co‐activation and global signal representation maps comparison

Voxel‐wise group differences were assessed with permutation testing and threshold‐free cluster enhancement using *FSL randomize* (Smith & Nichols, 2009; Winkler, Ridgway, Webster, Smith, & Nichols, 2014). Age, gender, and education level were included as covariates and the analyses were limited to gray matter voxels (*n* = 60′013) (Figure S3). Multiple comparison‐corrected statistical maps were thresholded at *p* < .05 to identify significant clusters (Winkler et al., 2014). Only clusters larger than 20 voxels were retained.

#### 2.7.2 Partial least square correlation between functional and clinical dimensions

Multivariate relationships between neuroimaging features (CAPs' occurrences) and clinical dimensions were assessed with partial least squares correlation (PLSC) analysis (Krishnan, Williams, McIntosh, & Abdi, 2011; Zöller et al., 2017) after forward step‐wise regression of age, gender, and education level confounds. PLSC identifies linear combinations of CAPs' occurrences and clinical scores with maximal covariance across subjects. Statistical significance of correlations was assessed with permutation testing (1,000 permutations); reliability of linear‐combination weights was assessed with bootstrapping (1,000 random samples with replacement) (Garrett, Kovacevic, McIntosh, & Grady, 2013; Kebets et al., 2019; Krishnan et al., 2011; Zöller et al., 2017).

## 3 RESULTS

### 3.1 Clinical characteristics

#### 3.1.1 iNPH patients perform less well than controls in gait and cognitive tests

iNPH patients were older, had lower education level and higher WM hypointensity load than HCs. Moreover, the iNPH group counted a smaller proportion of women. When correcting for age, gender, and education level, iNPH patients had worst gait performances than HCs, with decreased walking speed, increased step width, increased stride time, and shorter stride length. Patients had lower MMSE scores and performed less well in all cognitive domains: executive functions, memory, and attention. All statistical tests are reported in Table 1.

#### 3.1.2 Gait performances improve after CSF tap test

On average, gait improved after CSF tap test resulting in faster walking speed (63% of patients), lower stride time (73% of patients) and longer stride length (73% of patients) (Table 1, Figure S5). Among the cognitive functions, only one score of the attention tests improved on average after CSF tap test (WAIS‐III symbol digit modalities test, improved in 42% of patients).

### 3.2 Neuroimaging characteristics

#### 3.2.1 The default mode network is implicated in iNPH pathophysiology

Functional connectivity values within and between 17 RSNs were assessed for all subjects. The connectivity between the DMN and executive‐control network (ECN) (*F*[1,69] = 13.88, *p* = .0004, *d* = 1.08), and between the DMN and salience network (SAL) (*F*[1,69] = 15.32, *p* = .0002, *d* = 0.93) were increased in iNPH patients compared with HCs (comparisons surviving FDR‐correction). In general, connectivity between higher order systems (DMN, control, salience, and limbic circuits) tended to be increased, whereas connectivity between lower order systems (somatomotor and visual circuits, and limbic‐somatomotor connections) tended to be decreased in patients, but these comparisons did not survive FDR‐correction (Figure 1). Results were replicated using an alternative cortical parcellation (Figure S6).

#### 3.2.2 The precuneus has increased co‐activation with dorsolateral and medial frontal cortices

Individual precuneus co‐activation maps (Figure 2a) of patients and controls were compared at the voxel level for two contrasts: iNPH<HC; iNPH>HC. No gray matter voxel had decreased co‐activation with the precuneus seed in iNPH patients. Conversely, six voxel clusters had increased co‐activation with the precuneus (voxel‐level corrected *p* < .05). The three largest clusters (Clusters 1, 2, 3 in Figure 2c) correspond to the left and right dorsolateral prefrontal and medial frontal cortices. Smaller clusters extended over the left intraparietal sulcus (Cluster 4), left angular gyrus (Cluster 5) and right lateral frontal cortex (Cluster 6). Clusters size, MNI coordinates and *p*‐values are reported in Table S4.

#### 3.2.3 iNPH is characterized by abnormal dynamics measured by CAPs between the default mode, executive‐control, and salience‐attention networks

**FIGURE 3:**
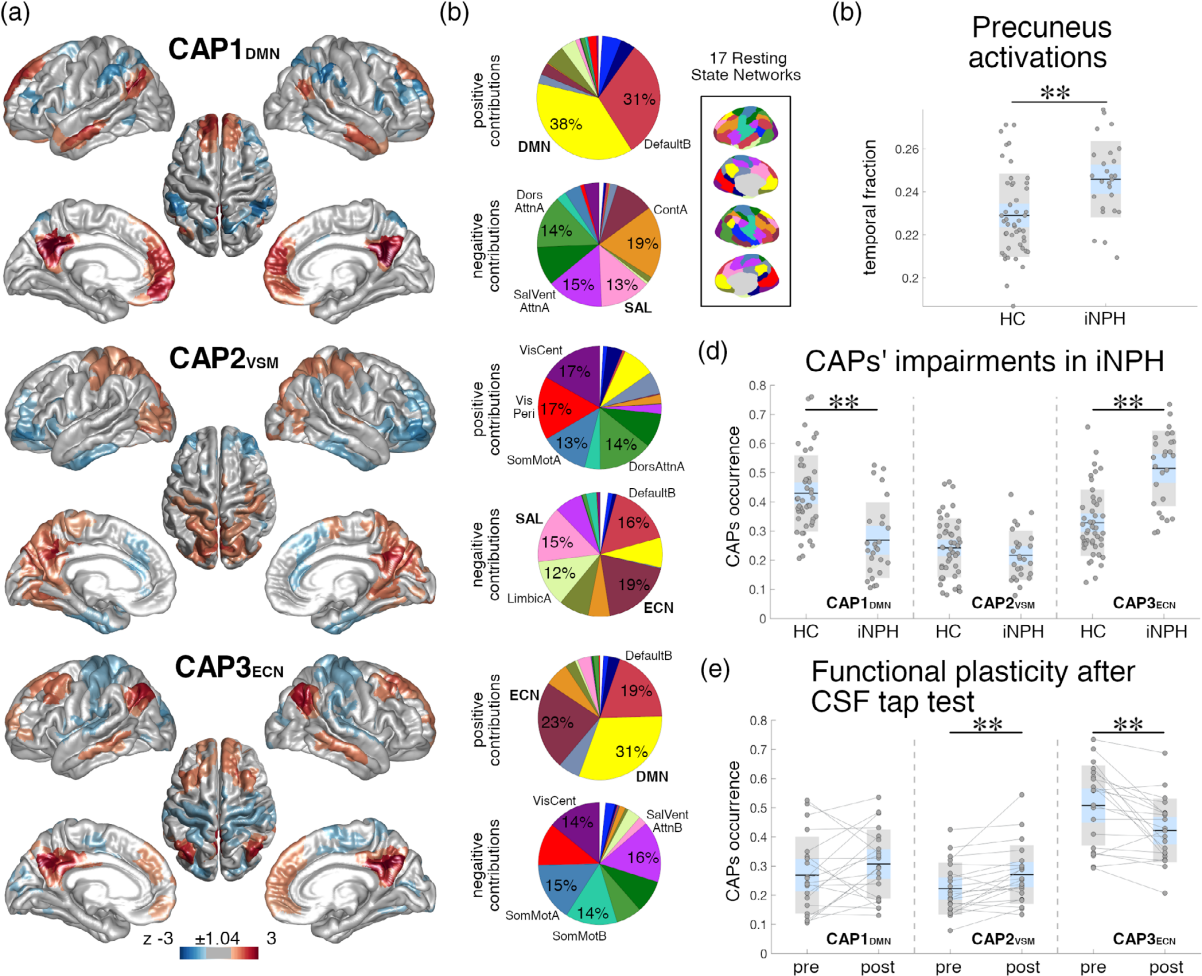
Co‐activation patterns (CAPs) are altered in iNPH and normalize after CSF tap test. (a) Z‐scored precuneus co‐activation patterns (CAPs) projected onto a standardized cortical surface. Only the 15% largest positive contributions and the 15% smallest negative contributions are represented in color. (b) Pie charts illustrating the percentage distributions of the positive and negative CAPs values over the 17 RSNs. The inset illustrates the 17 RSNs on an inflated cortical surface. (c) Fraction of fMRI volumes corresponding to precuneus activations in individual subjects of the iNPH and HC groups. (d) CAPs' occurrences in individual subjects, iNPH and HC groups. (e) CAPs' occurrences in iNPH patients assessed before (“pre”) and after (“post”) the CSF tap test. Gray segments link the same subjects in the pre‐ and post‐CSF tap test groups. Black segments and asterisks indicate statistically significant group‐comparisons (**p < .01). Boxplots: black lines indicate group means; light blue areas represent 95% confidence intervals; gray areas represent 1 *SD* intervals; raw data are jittered for better visualization

The fMRI volumes corresponding to precuneus activations were clustered into three CAPs encoding distinct functional configurations (Figure S7). The first CAP (“**CAP1**_**DMN**_”) exhibits a DMN activation pattern with co‐deactivation of the dorsal attention, ventral attention and salience networks (Figures 3a,b and S8). The second CAP (“**CAP2**_**VSM**_”) represents co‐activation of the precuneus with the visual, somatomotor and dorsal attention networks and co‐deactivation of higher order functional systems (executive‐control, salience, and limbic networks). The third CAP (**CAP3**_**ECN**_) reflects co‐activation of the DMN with the ECN and co‐deactivation with lower order functional systems (somatomotor, visual and ventral attention networks). iNPH patients had lower occurrence of **CAP1**_**DMN**_ (*F*[1,69] = 16.61, *p* = .0001, *d* = −1.23) and higher occurrence of **CAP3**_**ECN**_ (*F*[1,69] = 28.54, *p* = .000001, *d* = 1.56) (comparisons surviving FDR‐correction) (Figure 3d). Group‐comparisons of CAPs duration and frequency showed similar iNPH‐HC patterns, but with smaller effect sizes (Figure S9), so that in the following analyses we focused on CAPs' occurrences only. The number of fMRI volumes corresponding to precuneus activations was higher in iNPH compared with HCs (*F*[1,69] = 7.49, *p* = .008, *d* = 0.89) (Figure 3c). CAPs iNPH‐HC statistical comparisons remained significant when co‐varying for the fraction of precuneus activation volumes. Results were robust to variations of the number of clusters in the CAPs analysis (Figure S10). CAPs estimated from both patients and controls, or from controls only, had high spatial similarity (average Pearson's correlation *r*[60′011] = 0.98, *p* < 10^−11^, Figure S11). The occurrences of **CAP1**_**DMN**_ and **CAP3**_**ECN**_ were strongly negatively correlated across subjects (*r*(72) = −0.79, *p* < 10^−11^; iNPH: *r*(24) = −0.79, *p* < 10^−5^; HC: *r*(46) = −0.67, *p* < 10^−6^) indicating a within‐subject balancing effect of **CAP1**_**DMN**_–**CAP3**_**ECN**_ dynamics (Figure S12).

#### 3.2.4 CAPs' occurrences reflect gait and executive dimensions of iNPH

**FIGURE 4:**
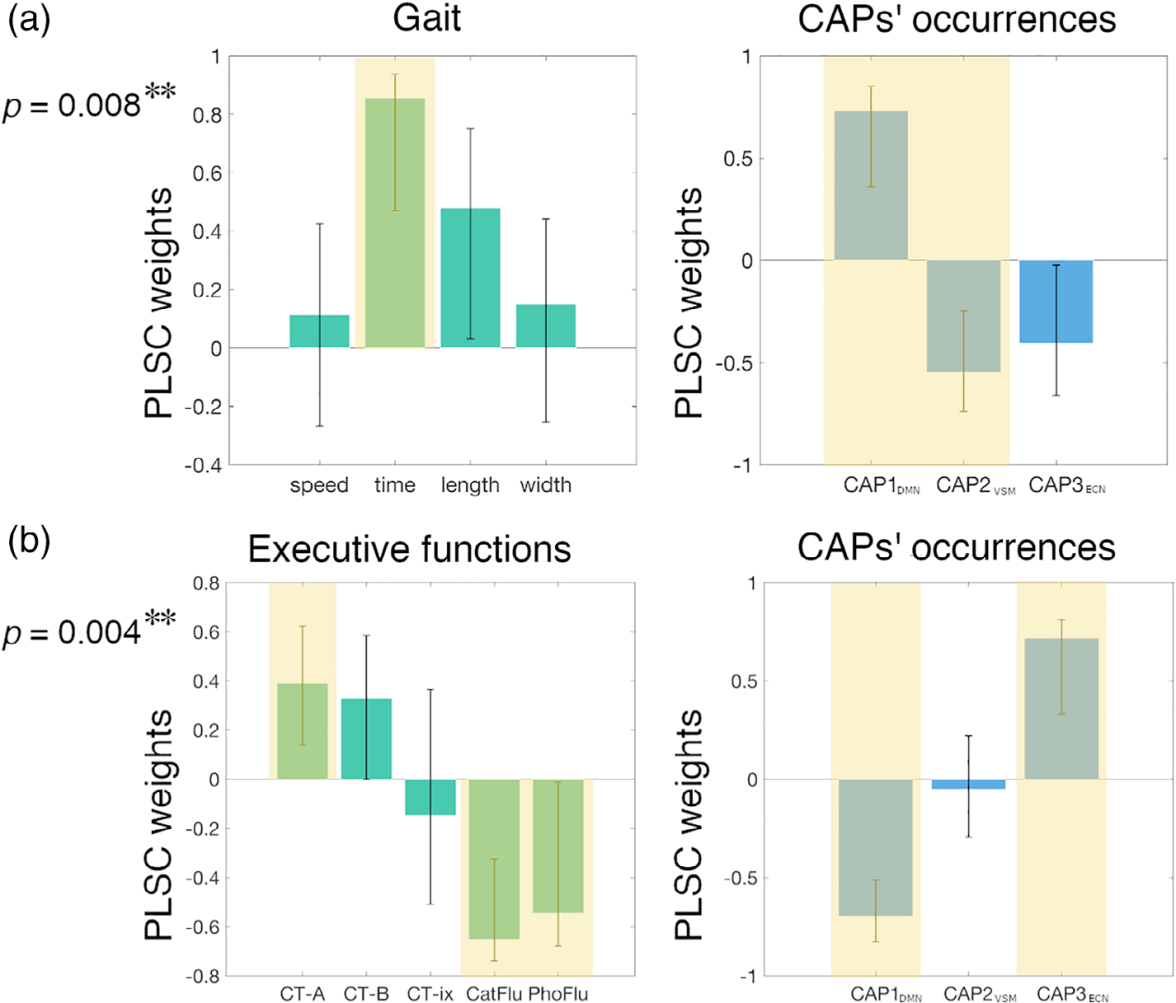
Correlation between CAPs' occurrences, gait and executive dimensions. Linear weights for the partial least squares correlation (PLSC) analyses on gait parameters and CAPs' occurrences (a), and executive scores and CAPs' occurrences (b). The PLSC linear weights represent the contributions of the different parameters to the maximal covariance pattern between gait/executive and neuroimaging dimensions. Error bars represent bootstrap intervals; yellow shadings highlight parameters with robust PLSC weights. *p*‐values refer to the significance of the PLSC analysis. **p < .01. Speed = walking speed; time = stride time; length = stride length; width = stride width; CT‐A = Color Trail test—Part A; Color CT‐B = Color Trail test—Part B; CT‐ix = Color Trail test—index; CatFlu = categorical verbal fluency; PhoFlu = phonemic verbal fluency

Four distinct PLSC analyses were run to assess the relationships between the three CAPs' occurrences and the gait, executive, memory, and attention domains in iNPH patients. These analyses revealed two significant correlations between the CAPs' occurrences, and the gait and executive domains (*p* = .004, *p* = .008, respectively; surviving FDR‐correction). Figure 4 illustrates the PLSC weights for the significant correlations. Worst (longer) stride time related to higher expression of **CAP1**_**DMN**_ and lower expression of **CAP2**_**VSM**_. Worst Color Trail–Part 1, semantic and phonemic fluency scores related to lower expression of **CAP1**_**DMN**_ and higher expression of **CAP3**_**ECN**_.

#### 3.2.5 CAPs' occurrences normalize after CSF tap test

FMRI volumes corresponding to precuneus activations were extracted from post‐CSF tap test data and classified in the three CAP‐clusters, established from the pre‐CSF tap test data as outlined above, based on Pearson's correlation values (Figure S13). The occurrence of **CAP2**_**VSM**_ was increased (*t*[20] = 3.43, *p* = .003, *d* = 0.75) and the occurrence of **CAP3**_**ECN**_ was decreased (*t*[20] = −3.64, *p* = .002, *d* = −0.79) after the CSF tap test, indicating a “partial normalization” of brain dynamics (Figure 3e). In particular, 81% of patients had higher **CAP2**_**VSM**_ occurrence and 81% had lower **CAP3**_**ECN**_ occurrence after the tap test. 67% of patients had higher **CAP1**_**DMN**_ occurrence after the tap test, but the comparison was not statistically significant (*t*[20] = 1.43, *p* = .17, *d* = 0.31).

#### 3.2.6 iNPH and tau pathology are associated with opposite CAPs dynamics

**FIGURE 5:**
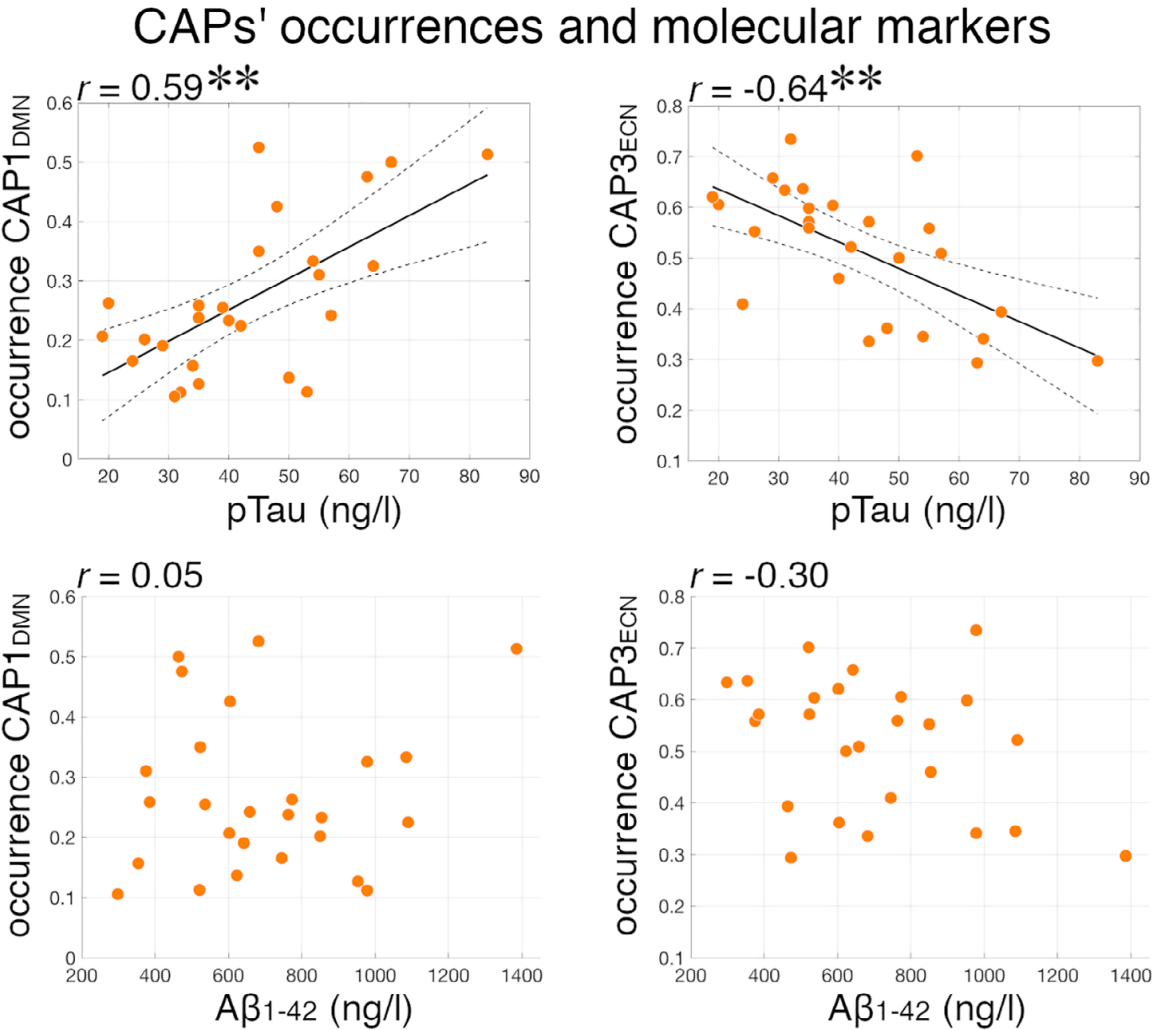
Association between CAPs' occurrences and Alzheimer's disease molecular markers. Scatter plots of pTau and A𝛽_1–42_ CSF levels versus **CAP1**_**DMN**_ and **CAP3**_**ECN**_ occurrences in iNPH patients. Pearson's correlation coefficients are reported above each scatter plots. The best linear fit (black line) and 95% confidence intervals (dashed lines) are reported for statistically significant correlations. **p < .01

In our cohort, 23% of patients were amyloid‐positive and 15% were tau‐positive. ANCOVA analyses of CAPs' occurrences were repeated including amyloid‐negative (*N* = 20) or tau‐negative (*N* = 22) patients only. All comparisons remained statistically significant for **CAP1**_**DMN**_, **CAP3**_**ECN**_ iNPH‐HC differences and **CAP2**_**VSM**_, **CAP3**_**ECN**_ pre‐/post‐CSF tap test differences (Table 2), indicating that AD amyloid and tau pathologies do not drive our results. Moreover, comparisons remained significant when co‐varying for WM hypointensities, an indicator of possible cerebrovascular WM comorbidity (Table 2). We tested the associations between A𝛽_1–42_ and pTau CSF levels, and CAPs' occurrences. There was a positive relationship between pTau levels and **CAP1**_**DMN**_ occurrence (*r*(24) = .63, *p* = .0006), and a negative relationship between pTau levels and **CAP3**_**ECN**_ occurrence (*r*(24) = −.67, *p* = .0002), but no association with A𝛽_1–42_ levels (Figure 5). These results suggest that iNPH and tau pathways are associated with distinct CAPs patterns: while iNPH patients have lower **CAP1**_**DMN**_ and higher **CAP3**_**ECN**_ occurrence compared with HCs, higher levels of pTau are associated with higher **CAP1**_**DMN**_ and lower **CAP3**_**ECN**_ occurrences in patients. There was no significant correlation between WM hypointensities and CAPs' occurrences in patients.

**TABLE 2:** Co‐activation patterns (CAPs) alterations and iNPH comorbidities

	CAP1_DMN_	CAP2_VSM_	CAP3_ECN_
	*p*‐value (effect size)	*p*‐value (effect size)	*p*‐value (effect size)
**iNPH—HC**			
All subjects (26 iNPH, 48 HCs)	**.0001 (−1.23)**	.28 (−0.27)	**.000001 (1.56)**
Amyloid‐negative patients (20 iNPH, 48 HCs)	**.0001 (−1.33)**	.48 (−0.16)	**.000002 (1.57)**
Tau‐negative patients (22 iNPH, 48 HCs)	**.000006 (−1.57)**	.35 (−0.26)	**<10e−7 (1.95)**
WM hypointensities covariate (26 iNPH, 48 HCs)	**.0002 (−1.23)**	.52 (−0.27)	**.00001 (1.56)**
**Pre–post CSF tap test**			
All subjects (21 iNPH)	.17 (0.31)	**.0027 (0.75)**	**.0016 (−0.79)**
Amyloid‐negative patients (17 iNPH)	.26 (0.28)	**.013 (0.68)**	**.011 (−0.70)**
Tau‐negative patients (18 iNPH)	.12 (0.38)	**.0083 (0.70)**	**.0023 (−0.85)**

*Note*: Co‐activation patterns changes in iNPH patients compared with HCs (assessed with ANCOVA), and in iNPH patients before and after the CSF tap test (assessed with paired *t* test). The table reports *p*‐values and Cohen's d effect sizes for all statistical tests. Group‐comparison were repeated on all available subjects; considering amyloid‐negative iNPH patients only; considering tau‐negative iNPH patients only; adding the WM hypointensities volume normalized by the intracranial volume as covariate. Statistically significant comparisons surviving FDR correction over the three CAPs tests are highlighted in bold. For the iNPH‐HC comparisons, positive (negative) effect sizes indicate increased (decreased) CAPs' occurrences in iNPH compared with HCs; for the pre–post CSF tap test comparisons, positive (negative) effect sizes indicate increased (decreased) CAPs' occurrences after the CSF tap test.

Finally, we explored the distribution of positive and negative amyloid and tau biomarkers with respect to the response to the CSF tap test. When considering the CAPs' occurrence changes after the CSF tap test, 17 of 21 patients (81%) had increased **CAP2**_**VSM**_ (all amyloid‐positive and all tau‐positive patients had increased **CAP2**_**VSM**_), and 17 of 21 (81%) had **CAP3**_**ECN**_ (only one tau‐positive patient did not have decreased **CAP3**_**ECN**_) occurrence. Fourteen of 21 patients (67%) had both increased **CAP2**_**VSM**_ and decreased **CAP3**_**ECN**_ occurrence (only one tau‐positive patients did not have both **CAP2**_**VSM**_ and **CAP3**_**ECN**_ changes) (Figure S14). Seventeen patients received quantitative gait assessments before and after the CSF tap test. When considering the gait changes after the CSF tap test, 12 of 17 patients (70%) had increased walking speed, and 14 of 17 (82%) had increased stride length. Only one amyloid‐positive patient had decreased walking speed and stride length. Twelve of 17 patients (70%), including the majority of the amyloid‐ and tau‐positive patients, had both increased walking speed and stride length (Figure S14). These distributions suggest that amyloid‐ and tau‐positivity do not impede brain functional changes or clinical response to CSF tap test in iNPH.

#### 3.2.7 Global signal

The group‐average global signal representation map is shown in Figure 2b: regions with highest global signal representation encompass occipital and posterior‐medial regions, in agreement with previous reports (Li, Bolt, et al., 2019b; Power et al., 2017). Individual global signal representation maps of patients and controls were compared at the voxel level for two contrasts: iNPH<HC; iNPH>HC. No gray matter voxel had decreased local representation of the global signal in iNPH patients. Conversely, three voxel clusters had increased representation of the global signal in iNPH patients (voxel‐level corrected *p* < .05). The three clusters encompass the left and right dorsolateral prefrontal and medial frontal cortices, with partial overlap with the regions presenting increased co‐activation with the precuneus (Figure 2d). Clusters size, MNI coordinates and *p*‐values are reported in Table S5.

The main analyses of this work were repeated while including the global signal as an additional regressor in the fMRI preprocessing pipeline. The effect sizes of the iNPH‐HC comparisons for the functional connectivity values within and between 17 RSNs, assessed with and without global signal regression, were significantly correlated (*r*[151] = .66, *p* < 10^−19^) (Figure S15). The iNPH‐HC and pre‐/post‐CSF tap test comparisons for the voxel‐wise precuneus co‐activation maps and CAPs' occurrences obtained from data with global signal regression were consistent with results obtained without global signal regression (Figures S16 and S17).

## 4 DISCUSSION

INPH is a prevalent but poorly understood neurological disorder. In this cohort of iNPH patients with mostly poor gait and cognitive functions, which are improved after CSF tap test, we demonstrate an alteration of higher order resting‐state network dynamics compared with normal aging, involving the default mode, executive‐control, and salience/attention systems. We show that these functional alterations underlie gait and executive disturbances in patients and are not driven by AD pathology or white matter changes. On the contrary, CSF tau levels relate to functional alterations opposite to iNPH. Finally, we demonstrate for the first time the presence of resting‐state functional MRI plasticity mechanisms 24 hr after a CSF tap test, with partial normalization of functional dynamics.

We followed a hierarchical analytical approach from whole‐brain connectivity to specific network‐dynamics investigation to characterize the functional circuits implicated in iNPH pathophysiology. The whole‐brain analysis pointed out a major implication of the DMN—confirming previous data (Khoo et al., 2016)—, with iNPH‐related hyper‐connectivity between the DMN, and the ECN and SAL (Figure 1). We then investigated the spatial and temporal connectivity features of the main DMN hub, located in the precuneus/posterior cingulate cortex (Greicius, Krasnow, Reiss, & Menon, 2003; Raichle, 2015), by assessing the precuneus average co‐activation maps, and co‐activation patterns (CAPs) dynamics (Liu et al., 2018). In line with the whole‐brain analysis, the precuneus demonstrated increased co‐activation with parts of the ECN (dorsolateral prefrontal and left parietal cortices) and SAL (dorsal anterior cingulate cortex) in iNPH (Figure 2). These findings can be better interpreted through the lens of the CAPs' dynamics framework: while the hyper‐connectivity between the DMN and the ECN can be explained by a higher temporal occurrence of **CAP3**_**ECN**_, the (static) hyper‐connectivity between the DMN and SAL is explained by a lower occurrence of **CAP1**_**DMN**_, which expresses the classic default mode pattern with activation of the medial posterior and anterior cingulate cortices, lateral parietal, temporal, and entorhinal regions and deactivation of the SAL and attention networks (Figure 3b) (Andrews‐Hanna, Smallwood, & Spreng, 2014; Raichle, 2015). We also note that the effect size of the iNPH‐HC CAPs' differences is larger than the effect size of the static functional connectivity differences, with 94% of patients having **CAP3**_**ECN**_ occurrence above the mean of the control group (*d* = 1.56). These considerations highlight the relevance of considering temporal features (besides static connectivity values) of resting‐state functional patterns to disentangle the specific consequences of iNPH on brain activity in the absence of task.

The DMN has previously been related to iNPH (Khoo et al., 2016; Ogata et al., 2017) as well as to AD (Greicius, Srivastava, Reiss, & Menon, 2004; Jones et al., 2016; Pievani, Filippini, van den Heuvel, Cappa, & Frisoni, 2014). A study found decreased functional connectivity within the DMN in 17 iNPH patients compared with 15 healthy controls, but other resting‐state networks were not taken into account (Khoo et al., 2016). Counterintuitively, within‐DMN connectivity increased with worsening of clinical symptoms in the same patients (Khoo et al., 2016). Our analyses show abnormal interactions between the precuneus and higher order cortical regions, but not between the precuneus and other regions of the DMN (Figure 2), suggesting that iNPH is mainly characterized by abnormal cross‐network dynamics involving the DMN, rather than by an intrinsic impairment of the DMN itself. This becomes particularly relevant when contrasting the DMN, **CAP1**_**DMN**_ and **CAP3**_**ECN**_ findings in iNPH with AD pathology. While AD is a frequent comorbid disease in iNPH and share similar cognitive and behavioral changes (Malm et al., 2013), AD has been consistently identified as a “DMN disorder.” First, the functional connectivity within the DMN is impaired in AD and preclinical AD (Badhwar et al., 2017; Jones et al., 2016; Pievani et al., 2014; Sheline & Raichle, 2013), and the precuneus shows decreased functional MRI activity matching PET hypometabolism (Greicius et al., 2004), consistent with the accumulation of amyloid in the DMN regions (Buckner et al., 2005). In contrast, within‐DMN connectivity and precuneus activity are not reduced in our iNPH cohort (Figures 2and 3c). Second, according to the cascading network failure and the prion‐like propagation models of misfolded beta amyloid and tau proteins (Jones et al., 2016; Ossenkoppele et al., 2019; Raj, Kuceyeski, & Weiner, 2012; Vogel et al., 2020; Zhou, Gennatas, Kramer, Miller, & Seeley, 2012), AD brain alterations are theorized to spread from the DMN to multimodal functional hubs of the brain network, including salience and executive regions (Crossley et al., 2014; Mišić et al., 2015). These mechanisms can result in impairments of the SAL and ECN and decreased DMN inter‐network connectivity in AD (Agosta et al., 2012; Brier et al., 2012; Li et al., 2019a). On the contrary, iNPH patients demonstrate increased DMN‐ECN coupling (increased occurrence of **CAP3**_**ECN**_). Taken together, these considerations suggest that AD and iNPH pathologies may relate to distinct functional connectivity alterations, with possible implications for differential diagnosis (Andersson, Rosell, Kockum, Söderström, & Laurell, 2017; Lu et al., 2020). This is supported by the fact that the observed CAPs alterations in iNPH are not referable to Aβ‐42 and pTau levels in the CSF (Table 2). Moreover, in our iNPH cohort higher pTau levels correlate with CAPs dynamics opposite to iNPH alterations; that is, with higher occurrence of **CAP1**_**DMN**_ (DMN‐SAL decoupling) and lower occurrence of **CAP3**_**ECN**_ (DMN‐ECN coupling) (Figure 5). Although there is no previous report on functional MRI and molecular markers in iNPH, this result is consistent with previous studies showing an association between CSF or PET tau and amyloid levels, and function connectivity in the DMN and between the DMN and SAL in cognitively normal elderlies (Schultz et al., 2017; Wang et al., 2013). It is unclear whether the absence of an association between functional features and Aβ‐42 levels in our iNPH cohort would generalize to a larger sample, and this point certainly deserves further investigation in the future.

We found that iNPH is characterized by decreased DMN‐SAL and increased DMN‐ECN interactions. Previous literature, and a longstanding debate on the effect of global signal regression in fMRI analyses, indicate that the global signal may partially influence the hierarchy of interactions between task‐positive (e.g., ECN, SAL) and task‐negative (DMN) networks (Murphy & Fox, 2017). Here, we showed that the functional alterations observed in iNPH patients are not driven by the global signal. Nonetheless, and as a complementary finding, we observed that the spatial representation of the global signal is modulated by iNPH pathology: iNPH patients show increased global signal representation in medial and lateral prefrontal cortices (Figure 2d). The global signal has been related to ongoing neural activity (Schölvinck et al., 2010; Turchi et al., 2018) and to changes in baseline glucose metabolism in the brain (Thompson et al., 2016). An increased global signal representation may therefore be interpreted as an increased baseline activity in the frontal lobe of iNPH patients, compatible with (ineffective) compensatory mechanisms in response to motor and executive deficits. However, this conclusion should be taken with caution. While recent studies have associated individual global signal topographies to behavioral traits (Li, Bolt, et al., 2019b) and neuropsychiatric pathologies (Scalabrini et al., 2020; Wang et al., 2019; Yang et al., 2014), it is well understood that the global signal contains artefactual contributions (Power et al., 2017) and research is needed to elucidate its interpretation.

The resting‐state fMRI alterations observed in this study are coherent with our knowledge on iNPH pathophysiological mechanisms and brain changes (Griffa et al., 2020; Keong et al., 2016; Siasios et al., 2016; Tarnaris et al., 2009). Ventricle enlargement and compression of periventricular tissues can engender a cascade of harmful events leading to damage of WM tracts crossing the area. Several of these tracts extend from frontal to parietal and temporal regions linking parts of the DMN, ECN, SAL, and attention networks (Calamante et al., 2013; Greicius, Supekar, Menon, & Dougherty, 2009; Makris et al., 2005; Tarun, Behjat, Bolton, Abramian, & Van De Ville, 2020; van den Heuvel, Mandl, Kahn, & Pol, 2009) and have been implicated in iNPH pathophysiology, which suggests a possible link between WM alterations and impairments of cross‐network dynamics. This hypothesis should be tested in future research using multimodal analyses. New methods, including diffusion weighted‐functional MRI imprinting, spatiotemporal networks and graph spectral approaches, have recently been proposed to map the white matter circuitries associated with specific (transient) functional patterns (Calamante, Smith, Liang, Zalesky, & Connelly, 2017; Deslauriers‐Gauthier et al., 2019; Griffa et al., 2017; Huang et al., 2018; Tarun et al., 2020). In particular, a study on healthy young adults has identified the WM connectivity maps corresponding to distinct precuneus CAPs (Tarun et al., 2020), with direct possible extensions to our results. The comparison between CAPs functional impairments, CAP‐specific white matter connectivity maps, and voxel‐wise information about WM microstructure could deliver insights linking reversible and irreversible micro‐scale neurobiological processes to large‐scale brain dynamics.

The observed iNPH functional alterations are consistent with the clinical presentation of the disorder (Picascia et al., 2016; Williams & Malm, 2016). A recent meta‐analysis of task‐based fMRI studies probing the convergence of gait, executive and urinary functions in healthy adults, identified the medial frontal, dorsolateral prefrontal, anterior insula, and parietal regions as cortical basins of vulnerability to iNPH (Griffa et al., 2020), with substantial overlap with our findings (Figure 2). It is also well understood that the interaction between the SAL, DMN, and ECN is central to the accomplishment of higher order functions and integration of internal and external stimulus‐driven mental processes (Goulden et al., 2014; Jilka et al., 2014; Menon, 2011; Menon & Uddin, 2010). In our iNPH cohort, SAL‐DMN‐ECN dynamics, summarized by the CAPs' occurrences, relate to executive functions. The PLSC analysis‐blind to iNPH‐HC group differences‐associates worst executive performances in iNPH to lower **CAP1**_**DMN**_ and higher **CAP3**_**ECN**_ occurrence, which underpins the pattern of functional impairments observed between iNPH and healthy controls (Figure 4). The functional alterations observed in patients may therefore partially explain their cognitive deficits. In healthy adults, the resting‐state temporal variability of the DMN‐ECN interactions predicts cognitive flexibility (Douw, Wakeman, Tanaka, Liu, & Stufflebeam, 2016) and a disrupted interaction between these networks correlates with lower cognitive performances in subjects with mild cognitive impairments (Chand, Wu, Hajjar, & Qiu, 2017). In a complex reasoning task, the functional coupling between ECN and SAL and decoupling with DMN increase as function of increased task complexity (Hearne, Cocchi, Zalesky, & Mattingley, 2015). In iNPH patients, a higher baseline DMN‐ECN coupling at rest by **CAP3**_**ECN**_ might reflect an inability of DMN‐ECN decoupling during executive tasks. However, we did not find any significant relationship between the CAPs' occurrences and the attention and memory domains.

Worse gait performances in iNPH patients were associated with higher occurrence of **CAP1**_**DMN**_ and lower occurrence of **CAP2**_**VSM**_, expressing the interaction between the precuneus and visual‐somatomotor areas (Figure 4). Alterations of functional connectivity between somatomotor and cortico‐subcortical regions have been associated with altered gait parameters in several neurodegenerative disorders, including Parkinson's and Alzheimer's diseases, although the role of distinct neuropathological mechanisms on gait phenotypes is still not clear (Allali et al., 2018a). The interaction between the DMN and somatomotor network is impaired in older compared with young adults, suggesting that DMN‐sensorimotor co‐deactivation may relate to gait impairments and fall risk in aging (Rodriguez‐Sabate, Morales, Sanchez, & Rodriguez, 2019). However, further research is needed to uncover possible overlaps (or differences) between the neural circuitry underlying gait changes in iNPH and other neurodegenerative disorders or physiological aging. The association between better gait performances and lower occurrence of **CAP1**_**DMN**_ is somehow counterintuitive, since the occurrence of **CAP1**_**DMN**_ is reduced in iNPH patients compared with controls. Considering that the **CAP1**_**DMN**_ and **CAP2**_**VSM**_ occurrences are inversely correlated, the higher occurrence of **CAP1**_**DMN**_ in patients with worse gait performance may be a balancing effect for the lower occurrence of **CAP2**_**VSM**_ in the same subjects. However, this remains a speculative hypothesis that needs further confirmation.

CSF withdrawal induces functional plasticity with a partial normalization of resting‐state brain dynamics (Figure 3e). Eighty‐one percentage of patients experienced a decreased occurrence of **CAP3**_**ECN**_ after the tap test indicating a normalization of the DMN‐ECN interaction. Moreover, **CAP2**_**VSM**_ occurrence was increased in a similar proportion of patients, despite the absence of significant differences at baseline. A previous study showed restored somatomotor activity during a finger‐tapping test in iNPH responders to CSF drainage, conceptually in line with our findings (Lenfeldt et al., 2011). Few studies investigated changes of EEG activity before and after CSF tapping or shunting, but their results are inconsistent (Aoki et al., 2013; Aoki et al., 2019; Sand, Bovim, & Gimse, 1994). In our cohort, patients improved in gait, but not in cognitive performances after CSF tap test, suggesting that resting‐state functional dynamics may recover quicker than cognitive functions. The validation of this hypothesis would require a longitudinal follow‐up of patients. Finally, it is interesting to note that amyloid‐ and tau‐positivity assessed in the CSF do not seem to prevent brain functional and gait changes in response to CSF tap test (Figure S14). This is in line with recent studies showing no differences of A𝛽_1–42_ CSF levels between iNPH responders and non‐responders to CSF tap test or shunting (Hamdeh et al., 2018). However, findings on AD biomarkers assessed with PET imaging are more controversial (Hiraoka et al., 2015; Jang et al., 2018).

There are limitations to the current study. First, our analyses focused on cortical circuits only, although iNPH pathophysiology is likely to also involve subcortical and cerebellar circuits. However, our choice was dictated by the intention of maximizing the robustness of results, by minimizing spatial normalization biases that can be severe in periventricular/subcortical regions. In the same line, we paid particular attention to the correction of head motion artifacts and excluded patients with mediocre‐quality MRI data. Second, even though we adopted a hierarchical analytical approach, the dynamic functional connectivity analysis was limited to the DMN interactions; future work should tackle whole‐brain network dynamics. Third, molecular markers were assessed in the CSF rather than in brain tissues. Although this is the first study showing a relationship between molecular markers and functional dynamics in iNPH patients, availability of PET data would allow a better understanding of the relationship between iNPH and amyloid/tau deposition topographies. The available sample size was too small to reliably assess the effect of amyloid and tau pathways on CSF tap test response. However, it should be noted that this was a representative clinical sample and not a dataset drawn from less specific databases comparing large versus small ventricles. Fourth, changes of cognitive performances in iNPH patients were assessed through replication of neuropsychological tests before and after CSF tapping and may therefore be affected by learning biases (Benedict et al., 2017; Calamia, Markon, & Tranel, 2013). The inclusion of a test–retest reliability assessment of neuropsychological tests in an iNPH sample could deliver a better picture of possible cognitive changes in response to treatment.

## 5 CONCLUSION

This study offers an in‐depth characterization of resting‐state dynamics in iNPH. Alterations of cross‐network interactions between the default mode, and the executive control and salience networks partially explain iNPH symptoms, tend to normalize after CSF tap test, and are distinct from AD features. These results may contribute to the development of iNPH biomarkers for differential diagnosis and to the improvement of iNPH clinical management.

## CONFLICT OF INTEREST DISCLOSURE

All the authors report no conflict of interest to disclose.

## Supporting information


**Appendix** **S1:** Supplementary Information

## Data Availability

The data that support the findings of this study are available from the corresponding author upon reasonable request and in accordance with the Institutional Review Board of the Geneva University Hospital. Software for the CAPs and PLSC analyses is available at https://c4science.ch/source/CAP_Toolbox [Bolton et al., 2019], https://miplab.epfl.ch/index.php/software/PLS [Kebets et al., 2019; Zöller et al., 2017].
