# Supplementary material for: Dynamic functional networks in idiopathic normal pressure hydrocephalus: Alterations and reversibility by CSF tap test
Source: Hum Brain Mapp. 2020 Dec 9;42(5):1485–502. doi: 10.1002/hbm.25308 (PMC7927299; doi:10.1002/hbm.25308)
Supplement: Supplementary file 1 — Appendix S1: Supplementary Information [file HBM-42-1485-s001.docx]

**Dynamic functional networks in idiopathic Normal Pressure Hydrocephalus: Alterations and reversibility by CSF tap test**

Running title: **FMRI of Normal Pressure Hydrocephalus**

Alessandra Griffa^1,2^, Giulia Bommarito^1,2^, Frédéric Assal^1^, François R. Herrmann^3^, Dimitri Van De Ville^2,4^, Gilles Allali^1,5^

1. Department of Clinical Neurosciences, Division of Neurology, Geneva University Hospitals and Faculty of Medicine, University of Geneva, Geneva, Switzerland

2. Institute of Bioengineering, Center of Neuroprosthetics, Ecole Polytechnique Fédérale De Lausanne (EPFL), Geneva, Switzerland

3. Department of Rehabilitation and Geriatrics, Geneva University Hospitals and University of Geneva, Geneva, Switzerland

4. Department of Radiology and Medical Informatics, University of Geneva, Geneva, Switzerland

5. Department of Neurology, Division of Cognitive & Motor Aging, Albert Einstein College of Medicine, Yeshiva University, Bronx, NY, USA

**Supporting Information**

**Supplementary Table 1.**

**Magnetization-prepared rapid acquisition gradient echo (MPRAGE) MRI parameters**

| TA (acquisition time) | 5:03 min |
| --- | --- |
| TE (echo time) | 2.4 ms |
| TR (repetition time) | 2200.0 ms |
| FA (flip angle) | 9 deg |
| FOV (field-of-view) | 230 x 230 x 167 mm |
| Voxel size | 0.8 x 0.8 x 0.8 mm |
| Slice thickness | 0.8 mm |
| Interslice gap | 0 mm |
| Slice orientation | sagittal |
| Phase encoding direction | A >> P |
| Parallel imaging method | GRAPPA (acceleration factor PE 2) |
| Fat suppression | none |
| B0 shimming mode | standard |
| B1 shimming mode | patient-specific |
| Brain coverage | whole-brain |

**Supplementary Table 2.**

**Multiband accelerated echo planar imaging (fMRI) MRI parameters**

| TA (acquisition time) | 10:43 min |
| --- | --- |
| TE (echo time) | 30.0 ms |
| TR (repetition time) | 1057.0 ms |
| FA (flip angle) | 47 deg |
| FOV (field-of-view) | 210 x 210 x 160 mm |
| Voxel size | 2.5 x 2.5 x 2.5 mm |
| Slice thickness | 2.5 mm |
| Interslice gap | 0 mm |
| Slice orientation | transversal |
| Phase encoding direction | A >> P |
| Measurements | 600 |
| Multi-slice mode | interleaved |
| Multi-band accelerator factor | 4 |
| Fat suppression | Fat sat. |
| B0 shimming mode | standard |
| B1 shimming mode | TrueForm |
| Brain coverage | whole-brain |

**Supplementary Table 3.**

**Double-echo gradient echo field map MRI parameters**

| TA (acquisition time) | 1:44 min |
| --- | --- |
| TE1 (echo time 1) | 4.49 ms |
| TE1 (echo time 2) | 6.95 |
| TR (repetition time) | 600.0 ms |
| FA (flip angle) | 60 deg |
| FOV (field-of-view) | 210 x 210 x 160 mm |
| Voxel size | 2.5 x 2.5 x 2.5 mm |
| Slice thickness | 2.5 mm |
| Interslice gap | 0 mm |
| Slice orientation | transversal |
| Phase encoding direction | R >> L |
| Fat suppression | none |
| B0 shimming mode | standard |
| B1 shimming mode | TrueForm |
| Brain coverage | whole-brain |

**Supplementary Table 4.**

**Brain regions with increased functional co-activation with the precuneus in iNPH compared to HC**

The table reports clusters size, MNI coordinates of local t-statistic maxima, and multiple-comparison corrected p-values for the clusters showing increased co-activation with the precuneus in iNPH patients compared to HCs (voxel-level corrected *p* < 0.05). Clusters including less than 20 voxels are not reported. The table also reports the anatomical regions, overlapping resting state networks (Schaefer et al., 2018) and first results of Neurosynth searches corresponding to the significant clusters (Yarkoni, Poldrack, Nichols, Van Essen, & Wager, 2011).

| **Cluster** | **Size**  **(voxels)** | **MNI**  **coordinates** | **Smallest**  ***p*-value** | **Anatomical**  **label** | **Overlapping**  **RSN** | **Neurosynth**  **association** |
| --- | --- | --- | --- | --- | --- | --- |
| 1 | 462 | -43,50,12 | 0.002 | Left dorsolateral  prefrontal cortex | SalVentAttnB,  ContB,  ContA | Memory,  Recall,  Working memory |
| 2 | 246 | 44,48,7 | 0.018 | Right dorsolateral  prefrontal cortex | SalVentAttnB,  ContB,  ContA | Memory,  Recall,  Working memory |
| 3 | 228 | -1,23,52 | 0.013 | Bilateral medial  frontal cortex / dorsal anterior cingulate | SalVentAttnB | Monitoring,  Decision |
| 4 | 112 | -48,-49,49 | 0.013 | Left intraparietal sulcus | ContA | Working memory,  Memory retrieval,  Calculation |
| 5 | 57 | -28,-69,42 | 0.034 | Left angular gyrus | ContA | Working memory,  Memory retrieval,  Calculation |
| 6 | 29 | 29,10,62 | 0.036 | Right frontal lobe | ContB,  DefaultA | Working memory,  Attention |

**Supplementary Table 5.**

**Brain regions with increased global signal representation in iNPH compared to HC**

**The table reports clusters size, MNI coordinates of local t-statistic maxima, and multiple-comparison corrected p-values for the clusters showing increased global signal representation in iNPH patients compared to HCs (voxel-level corrected *p* < 0.05). Clusters including less than 20 voxels are not reported.**

| **Cluster** | **Size**  **(voxels)** | **MNI**  **coordinates** | **Smallest**  ***p*-value** |
| --- | --- | --- | --- |
| **1** | **981** | **24,18,57** | **0.003** |
| **2** | **394** | **-36,30,44** | **0.010** |
| **3** | **62** | **-31,63,7** | **0.016** |

**Supplementary Figure 1.**

**Cortical parcellation and resting state network labels**

The cortical volume of each subject was subdivided in 100 regions of interests (Schaefer et al., 2018) organized in 17 resting state networks (RSNs) (Yeo et al., 2011). A. Randomly color-coded regions of interest. B. Regions of interest color-coded according to the 17 resting state networks. In particular, the yellow, light pink and brown colors indicate the default mode (DMN), salience (SAL) and executive control (ECN) networks. C. Color-coded legend for the 17 resting state networks represented in panel B.

**
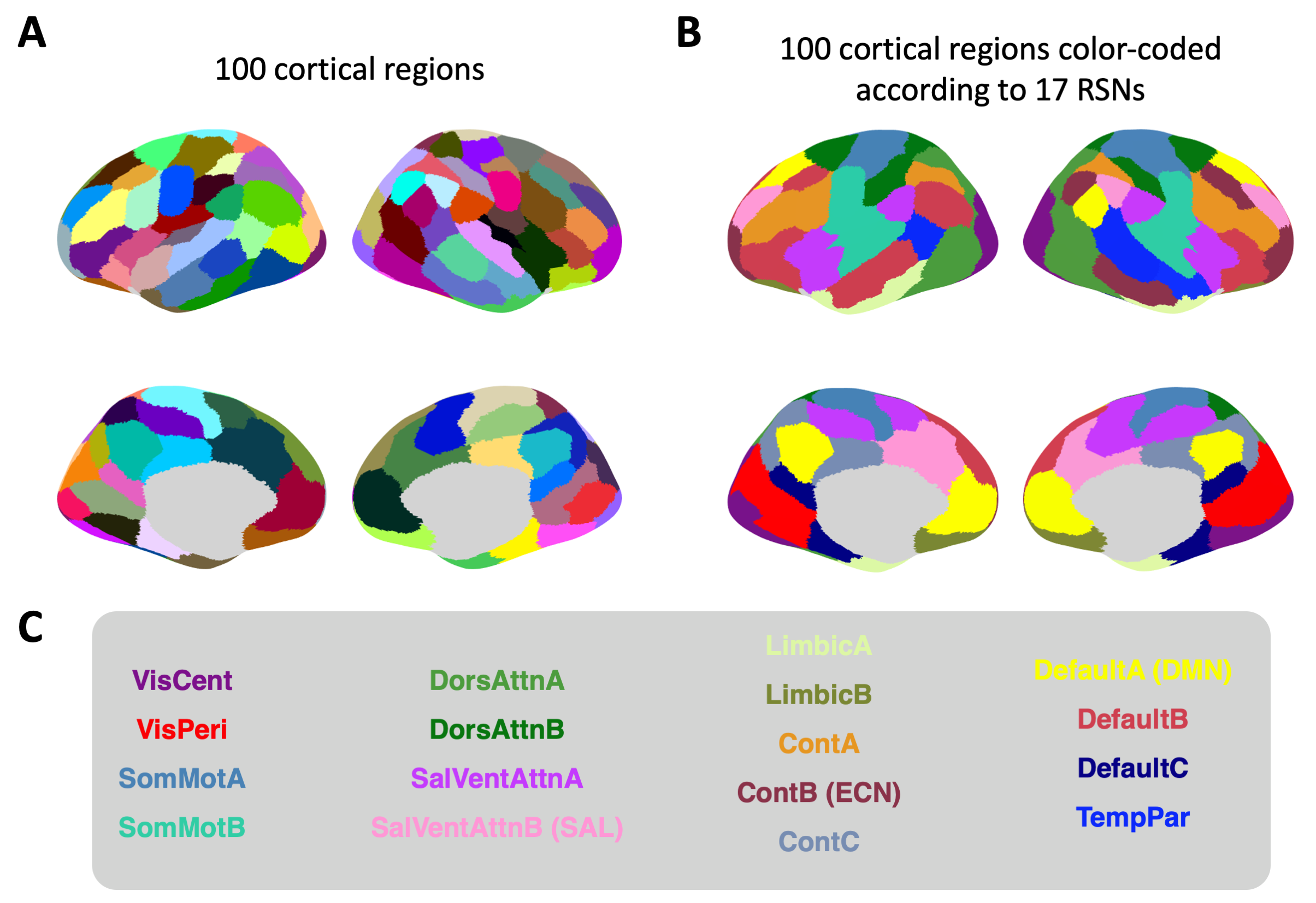
**

**Supplementary Figure 2.**

**Alternative cortical parcellation with 400 cortical regions**

Functional connectivity analysis were replicated using an alternative parcellation of 400 regions of interest grouped in 17 resting state networks (Schaefer et al., 2018; Yeo et al., 2011). A. Randomly color-coded 400 regions of interest. B. 400 Regions of interest color-coded according to the 17 resting state networks (RSNs).


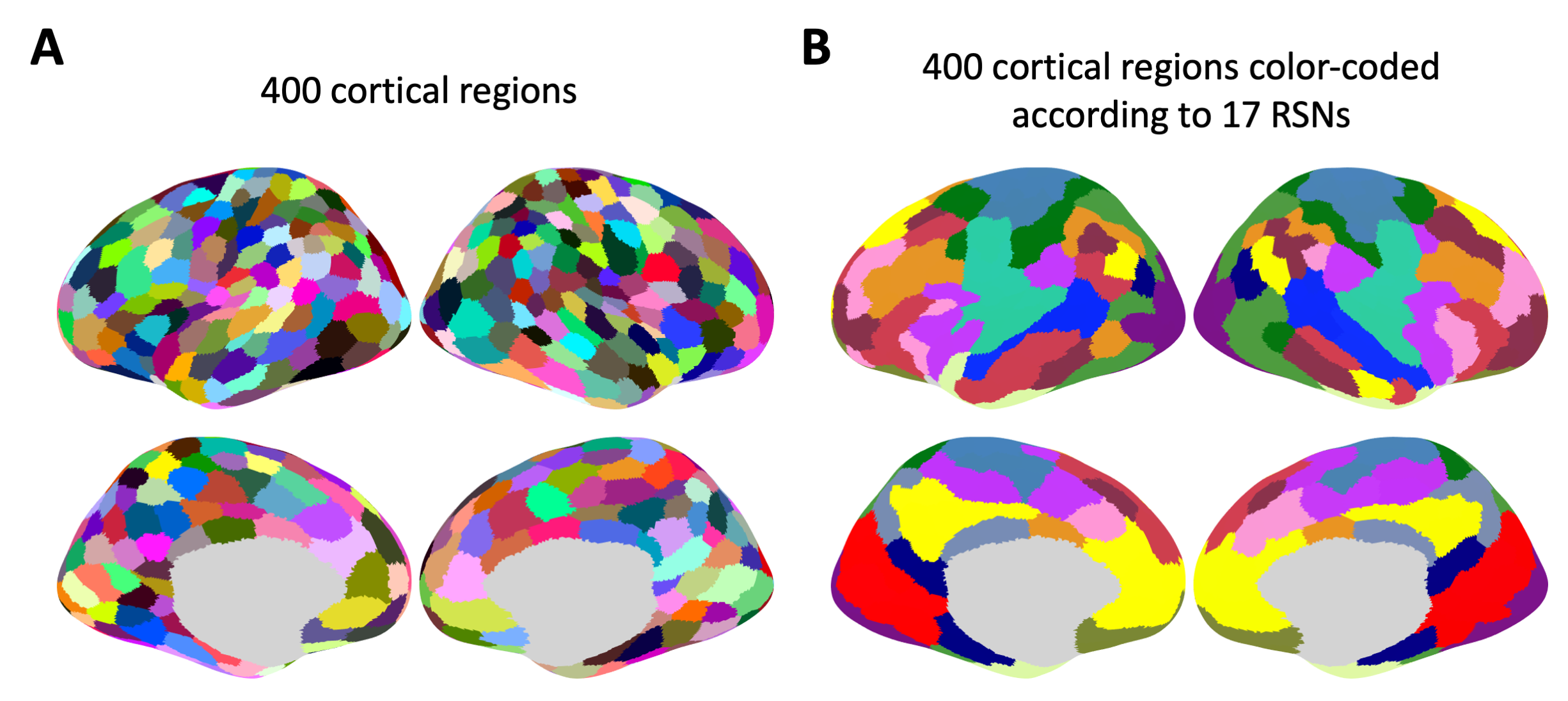


**Supplementary Figure 3.**

**Precuneus seeds for the co-activation maps analysis**

The precuneus seeds were defined by placing two spheres of radius 6.25 mm in the centroids of the left and right medial posterior components of the default mode network, indicated with two asterisks in panel A. B. Left and right precuneus (PCUN) seeds overlaid on the T1-weighted MNI reference volume (MNI ICBM 152 Nonlinear Asymmetrical template, version 2009c (Fonov, Evans, McKinstry, Almli, & Collins, 2009)). The orange transparency shows the cortical mask used for the voxel-wise precuneus co-activation maps group-comparison and includes 60’013 voxels.


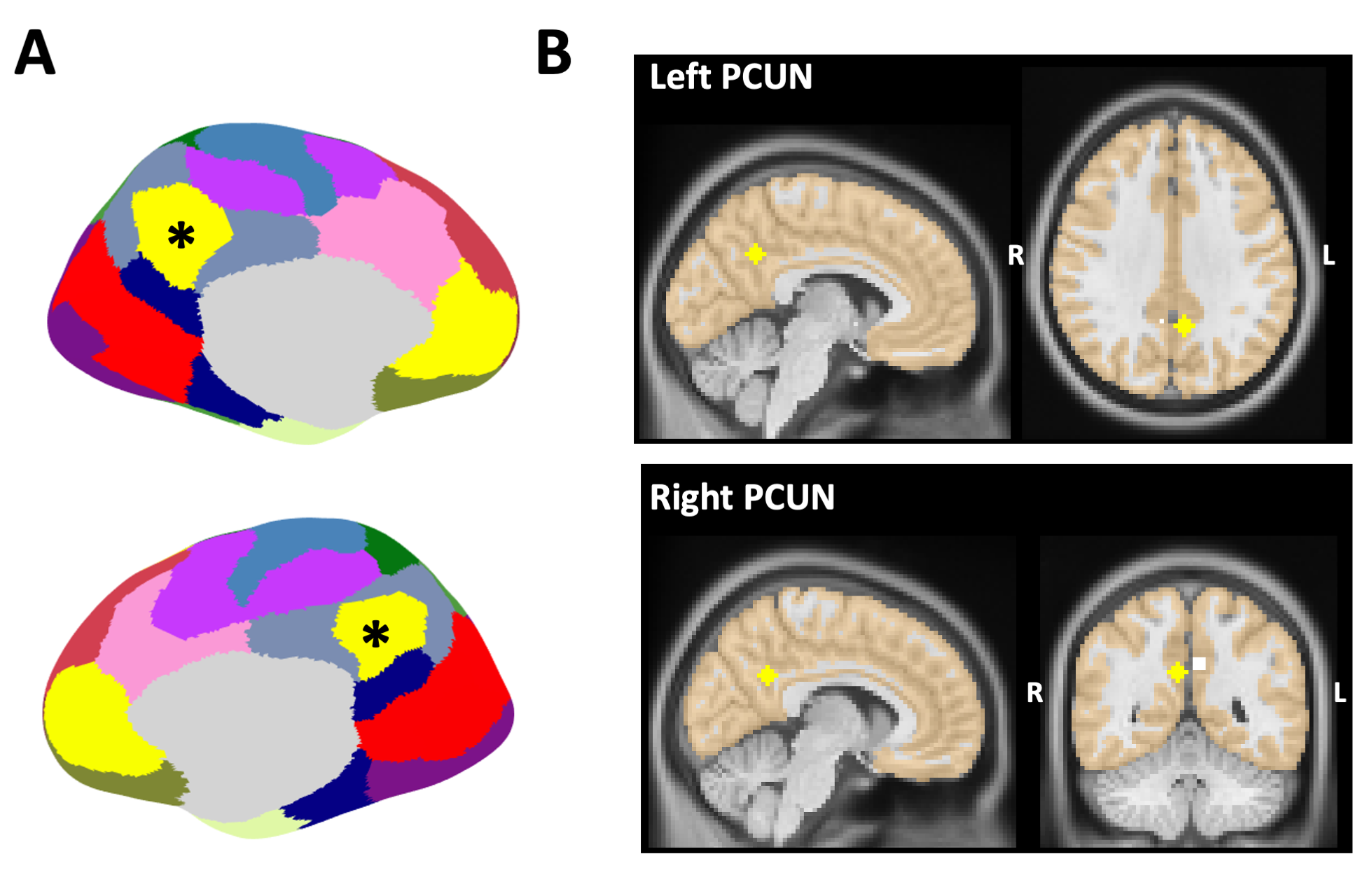


**Supplementary Figure 4.**

**Selection of the number of clusters for the precuneus co-activation pattern (CAP) analysis**

The number of cluster *k* was defined according to two criteria. First, we run a consensus clustering procedure for different values of *k* (*k* = 2:12) to assess the clusters robustness to small variations of the input dataset (Bolton et al., 2019; Monti, Tamayo, Mesirov, & Golub, 2003). In more detail, for each *k* the *k*-means clustering was run 20 times on 80%-folds of bootstrapped data with no replacement, and a consensus clustering matrix was computed from the 20 runs. Consensus clustering matrices for *k* = 2, 3, 4, 5 are shown in panel A; the color scale represents the percentage of time two data points are consistently classified in the same cluster. A robust clustering output would ideally result in a binary consensus clustering matrix with entries equal to 1 or 0 (i.e., two data points are always or never classified in the same cluster over the 20 runs), and in a stepwise cumulative density function of the consensus clustering matrix entries. According to these criteria, the highest consensus clustering robustness is achieved with *k* = 3. The empirical cumulative density functions (CDF) for *k* = 2:12 are represented in panel B. As a second criteria, we reasoned that a good number of clusters *k* would result in precuneus co-activation patterns (CAPs) consistently expressed across all the subjects (including iNPH patients and healthy controls). Panel B represents the minimum (orange line) and 5-percentile (black line) numbers of data points assigned to a CAP-cluster in individual subjects. A number of clusters *k* > 4 would result in CAPs that are not expressed in all the subjects.


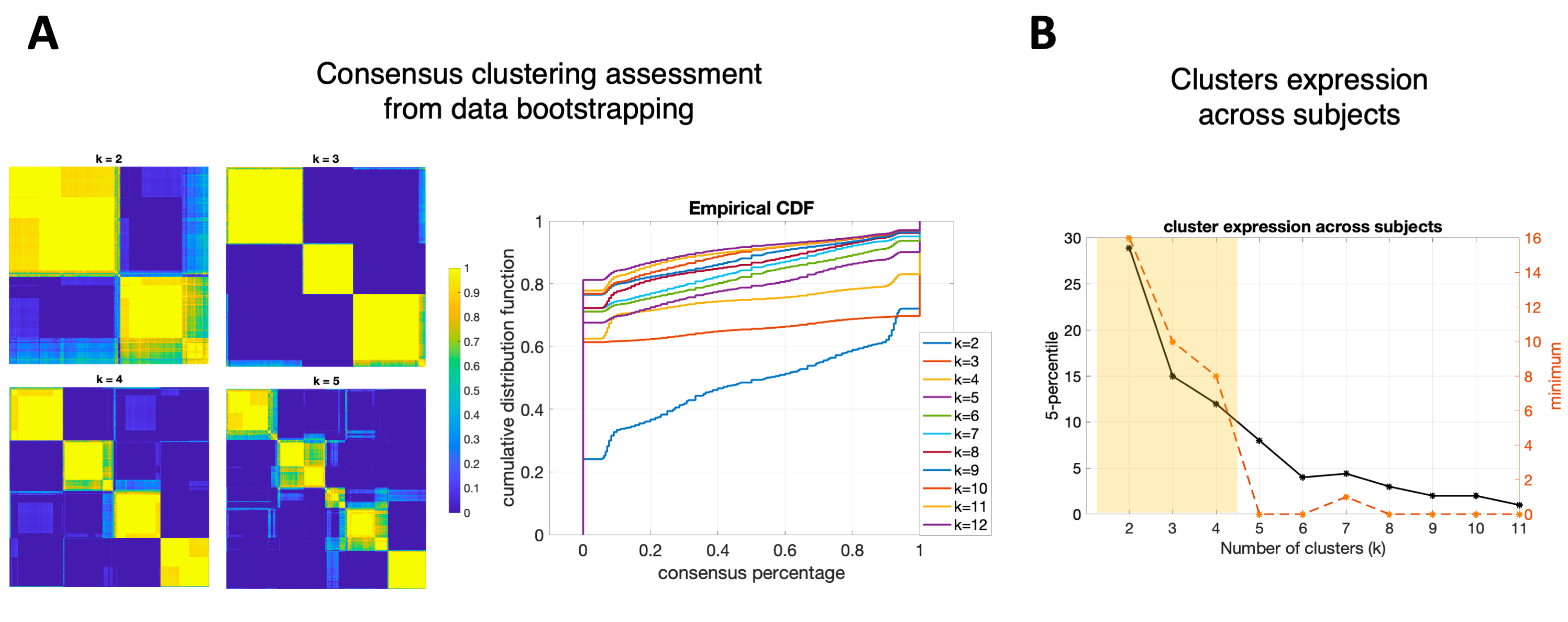


**Supplementary Figure 5.**

**Pre- / post-CSF tap test changes of gait and attention scores**

Percentual change of gait and attention scores between pre-CSF tap test assessment and post-CSF tap test assessment for 21 iNPH patients. The percentual change was computed as the post-CSF tap test score minus the pre-CSF tap test score, divided by the pre-CSF tap test score. Negative stride time, positive stride length, and positive WAISS-III percentual changes indicate an improvement after the CSF tap test. Grey spherical markers indicate missing values.


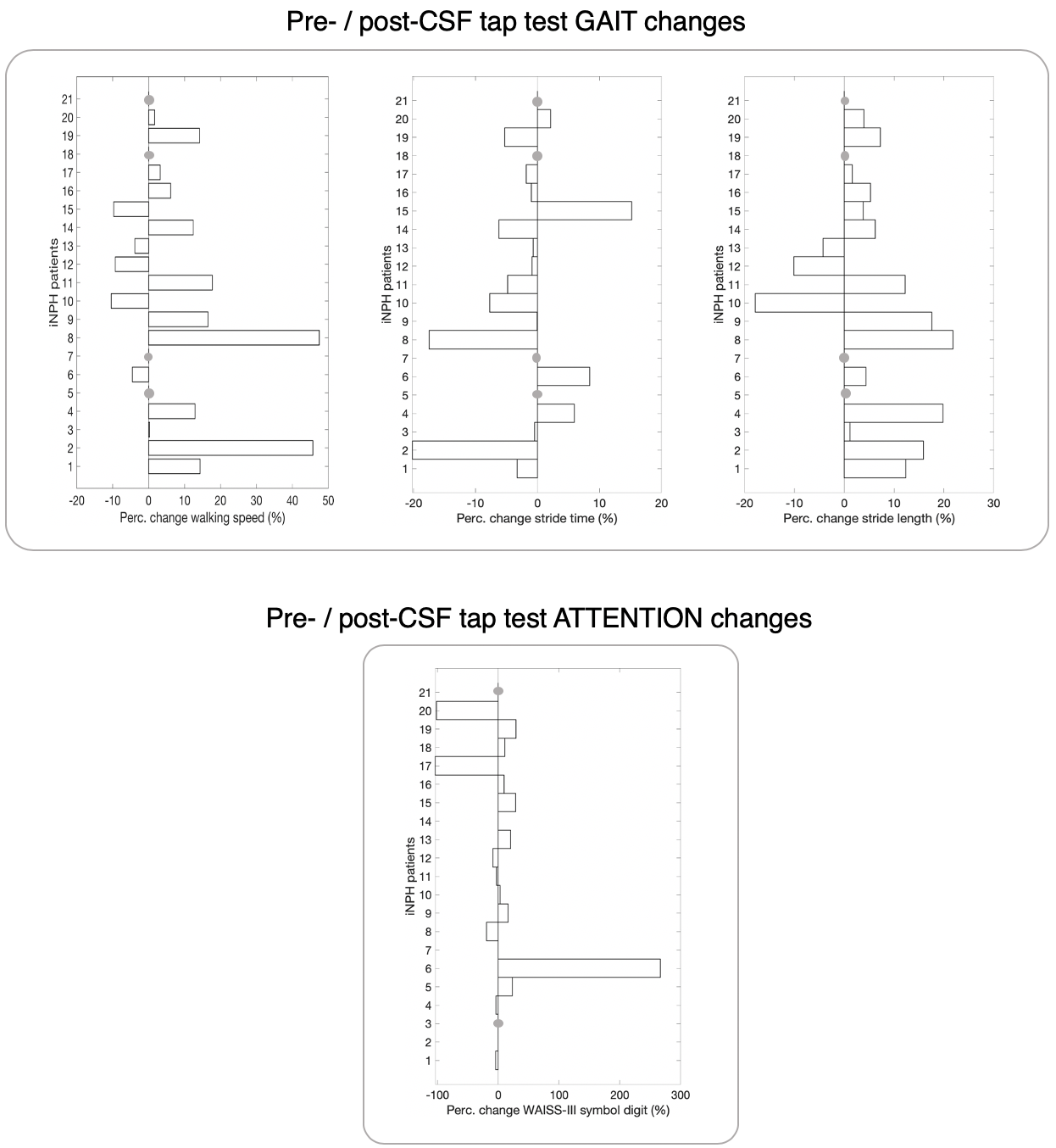


**Supplementary Figure 6.**

**Resting-state network connectivity in iNPH patients and healthy controls with alternative parcellation**

Functional connectivity values within- and between-resting state networks (RSNs) were computed using and alternative cortical parcellation including 400 regions of interest grouped in 17 RSNs (Schaefer et al., 2018; Yeo et al., 2011). Results were consistent with the main analysis, indication a major involvement of the default mode network (‘DefaultA’). The two matrices represent the p-values (ANCOVA) and Cohen’s *d* effect sizes for the iNPH (pre-CSF tap test assessment)-HC group-comparisons of functional connectivity values.


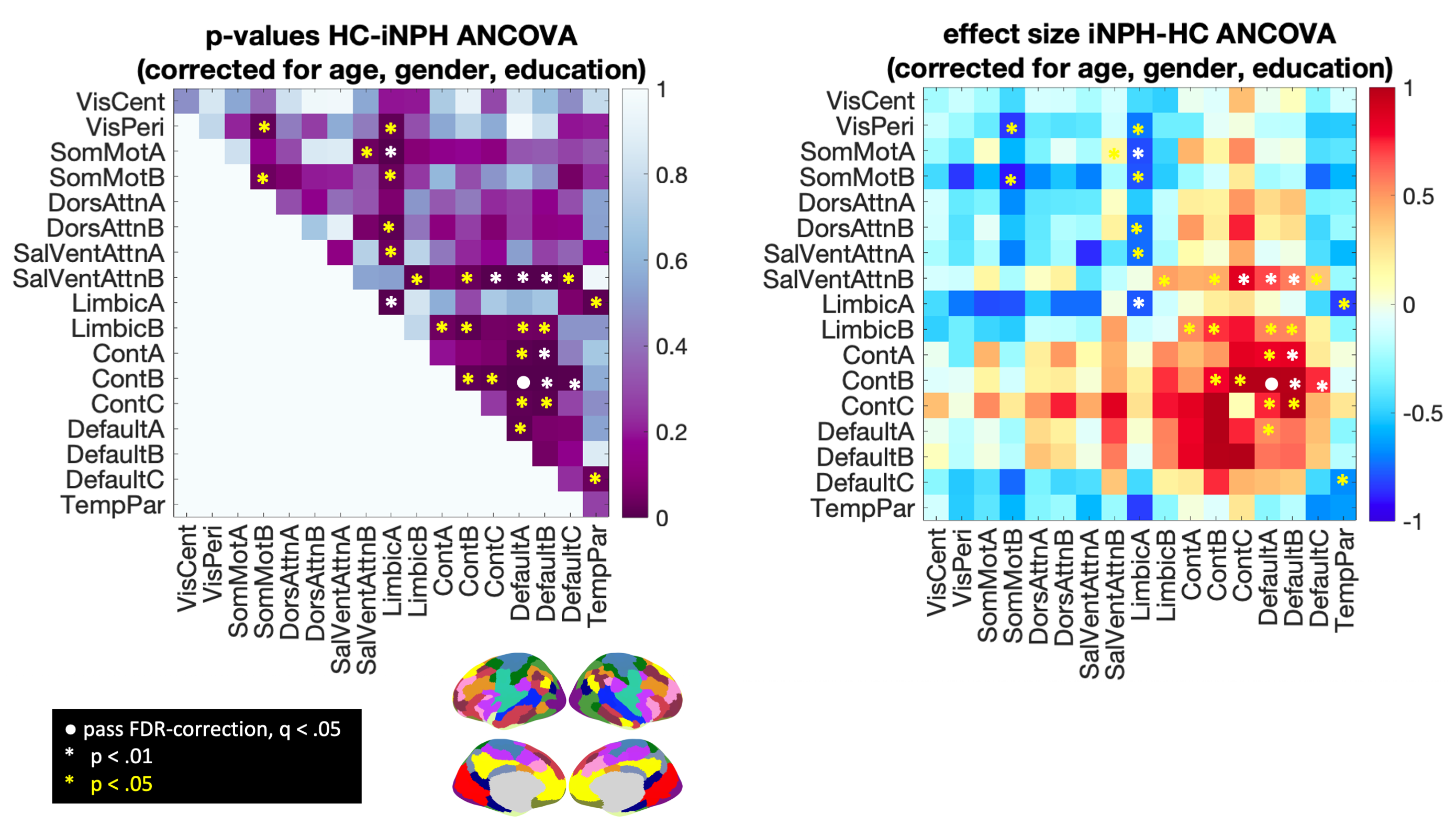


**Supplementary Figure 7.**

**Correlations between CAPs**

Pearson’s correlation values between the three precuneus CAPs. Medium-low correlation values between CAPs indicate that the CAPs encode distinct coactivation patterns.


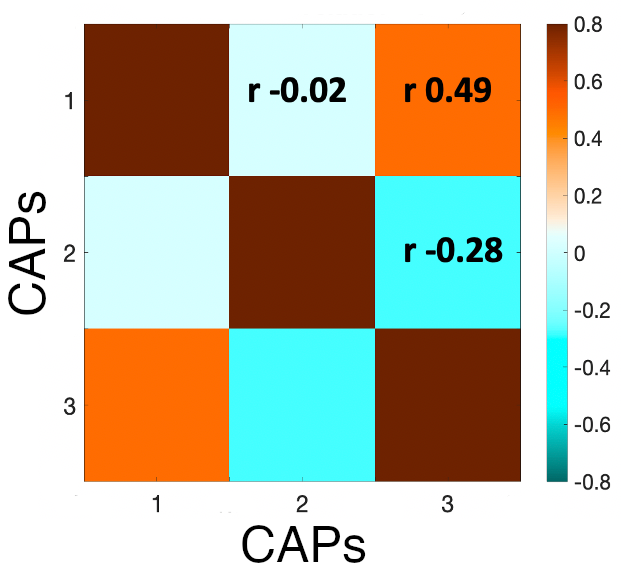


**Supplementary Figure 8.**

**Precuneus co-activation patterns (CAPs)**

Z-scored precuneus cortical CAPs overlaid onto the MNI T1-weighted template (MNI ICBM 152 Nonlinear Asymmetrical template, version 2009c (Fonov et al., 2009)). Voxels with positive (negative) z-scored values represent co-activations (co-deactivations) with the precuneus seed. The normalized averages of the positive and negative voxel-wise CAPs values within 17 resting state networks (RSNs) represent the positive and negative percentual contributions of the RSNs to each CAP (pie charts in Fig. 3B of the main manuscript). Positive contributions of CAP1**_DMN_** extend over the superior frontal and rostral anterior cingulate, inferior parietal and middle temporal cortices, while negative contributions involve the superior parietal, lateral prefrontal, pars opercularis, temporo-occipital and anterior insula cortices. Positive contributions of CAP2**_VSM_** involve the precentral and postcentral gyri, calcarine, extrastriate and auditory cortices, while negative contributions include the lateral prefrontal, dorsal anterior cingulate, supplementary motor area, inferior temporal and entorhinal cortices. Positive contributions of CAP3**_ECN_** cover the inferior parietal lobule/intraparietal sulcus, superior frontal, caudal middle frontal and middle temporal cortices, while negative contributions include precentral and postcentral gyri, posterior paracentral lobule, visual and auditory cortices.


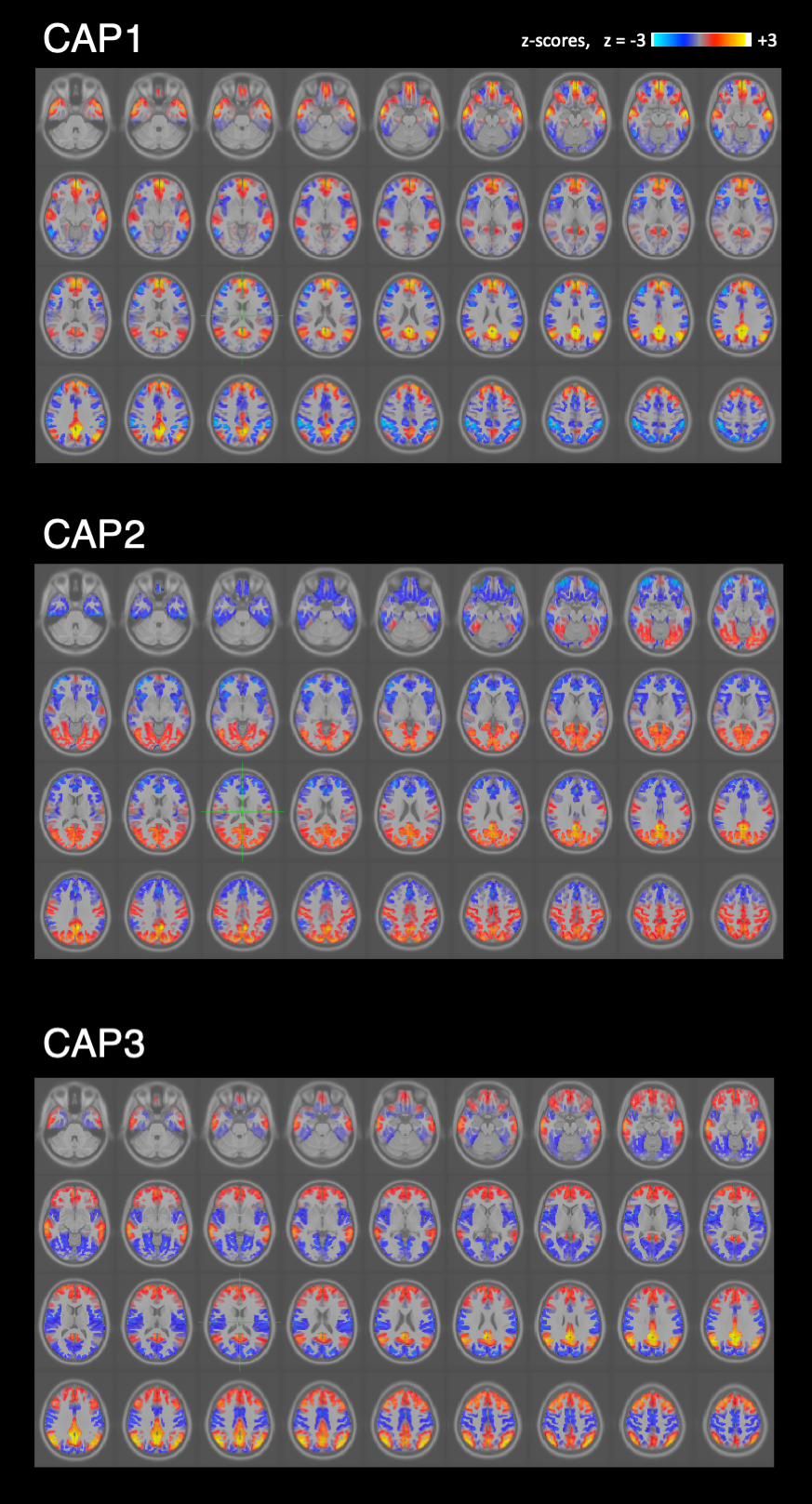


**Supplementary Figure 9.**

**CAPs duration and frequency for iNPH patients and healthy controls**

A. CAPs frequencies (CAP occurrences / s). B. CAPs durations (s). P-values from iNPH-HC ANCOVA analyses are reported. * group-differences surviving multiple comparison correction. Box-plots: black lines indicate group means; light blue areas represent 95% confidence intervals; grey areas represent 1 standard deviation intervals; raw data are jittered for better visualization.

**
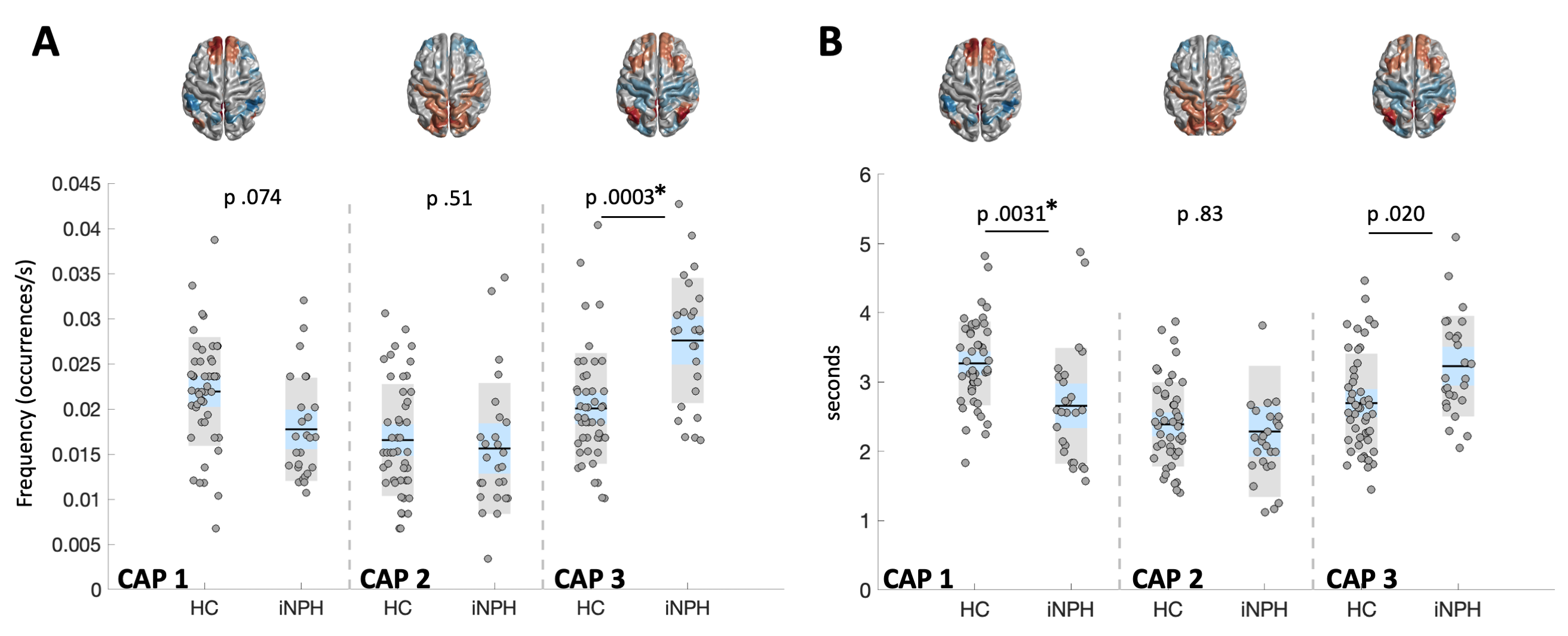
**

**Supplementary Figure 10.**

**Replication of main CAPs analyses with a different number of clusters (*k* = 4).**

A. Z-scored precuneus co-activation patterns (CAPs) projected onto a standardized cortical surface, obtained from the *k*-means clustering of precuneus activation frames when setting *k* = 4. Only the 15% largest positive contributions and the 15% smallest negative contributions are represented in color (light to dark red: 1.04<z<3; light to dark blue: -3<z<-1.04). We note that CAP1 and CAP2 express the co-activation of the DMN with the ECN; CAP3 represents a DMN activation with co-deactivation of the SAL and attention networks; CAP4 represents the interaction of the precuneus with somatomotor, visual and attention regions. B. Pearson’s correlation coefficients between the 4 CAPs. C. CAPs occurrences in individual subjects, for the iNPH and HC groups. P-values from iNPH-HC ANCOVA analyses are reported. * group-differences surviving multiple comparison correction. Box-plots: black lines indicate group means; light blue areas represent 95% confidence intervals; grey areas represent 1 standard deviation intervals; raw data are jittered for better visualization.

**
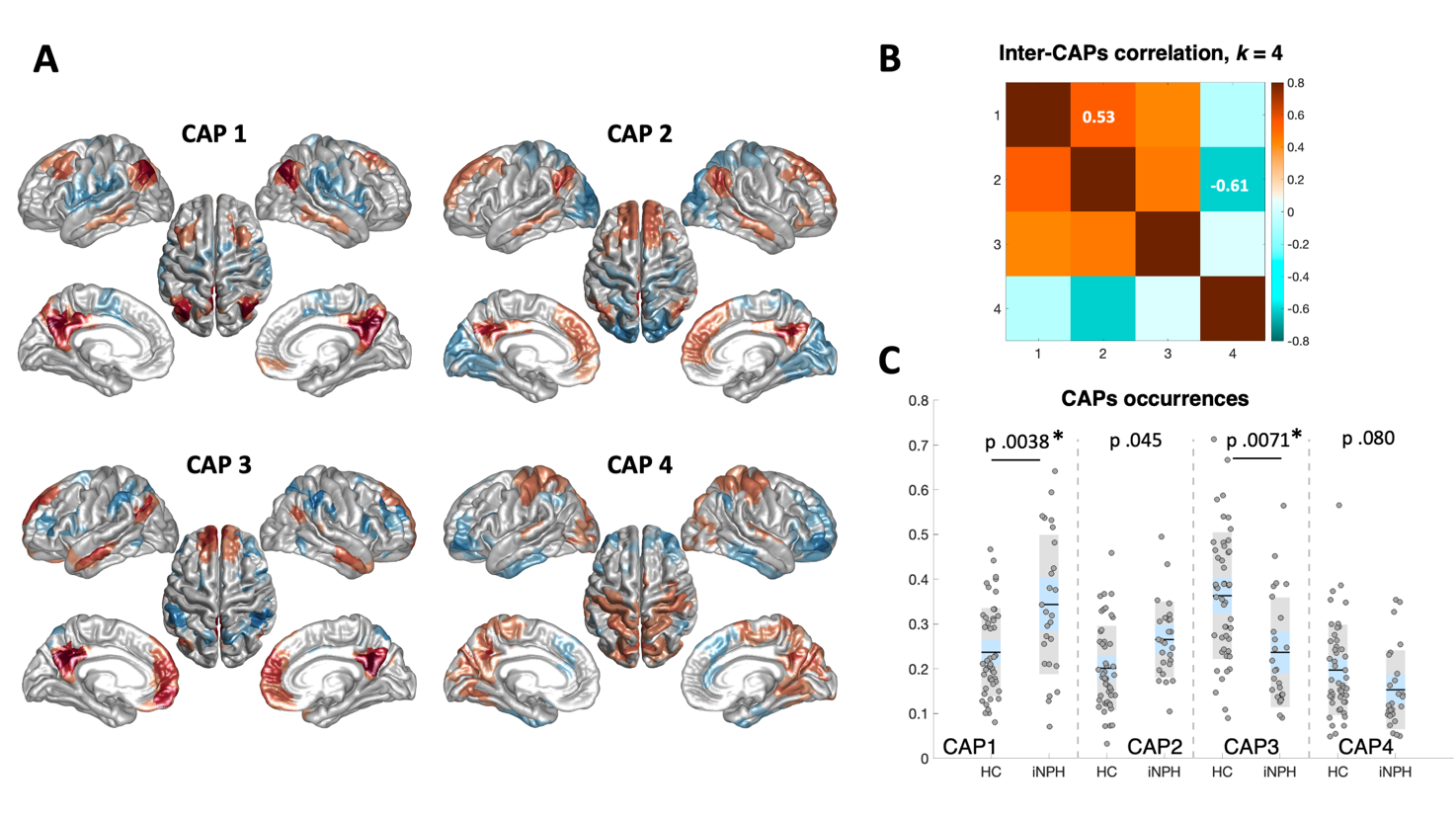
**

**Supplementary Figure 11.**

**CAPs estimated from healthy controls only.**

A. Z-scored precuneus co-activation patterns (CAPs) projected onto a standardized cortical surface, obtained from the *k*-means clustering of precuneus activation frames of healthy controls (HCs) only. The 15% largest positive contributions and 5% smallest negative contributions are represented in color (light to dark red: 1.04<z<3; light to dark blue: -3<z<-1.04). B Pearson’s correlation coefficient between the CAPs estimated from the HCs (rows) and the CAPs jointly estimated from the HCs and iNPH patients (columns). We observe a high similarity between the CAPs estimated on the two datasets. B. Histograms of the Pearson’s correlation coefficients between the single fMRI frames corresponding to precuneus activations, and the closest CAP, for iNPH patients (orange bars) and HCs (blue bars). We observe a consistent overlap between the two histograms, indicating that the CAPs are equally representative of the precuneus functional patterns occurring in both iNPH patients and HCs. D. iNPH-HC CAPs occurrences comparisons when using the CAPs templates estimated on HCs only. Significant p-values from ANCOVA analyses are reported. Box-plots: black lines indicate group means; light blue areas represent 95% confidence intervals; grey areas represent 1 standard deviation intervals; raw data are jittered for better visualization.


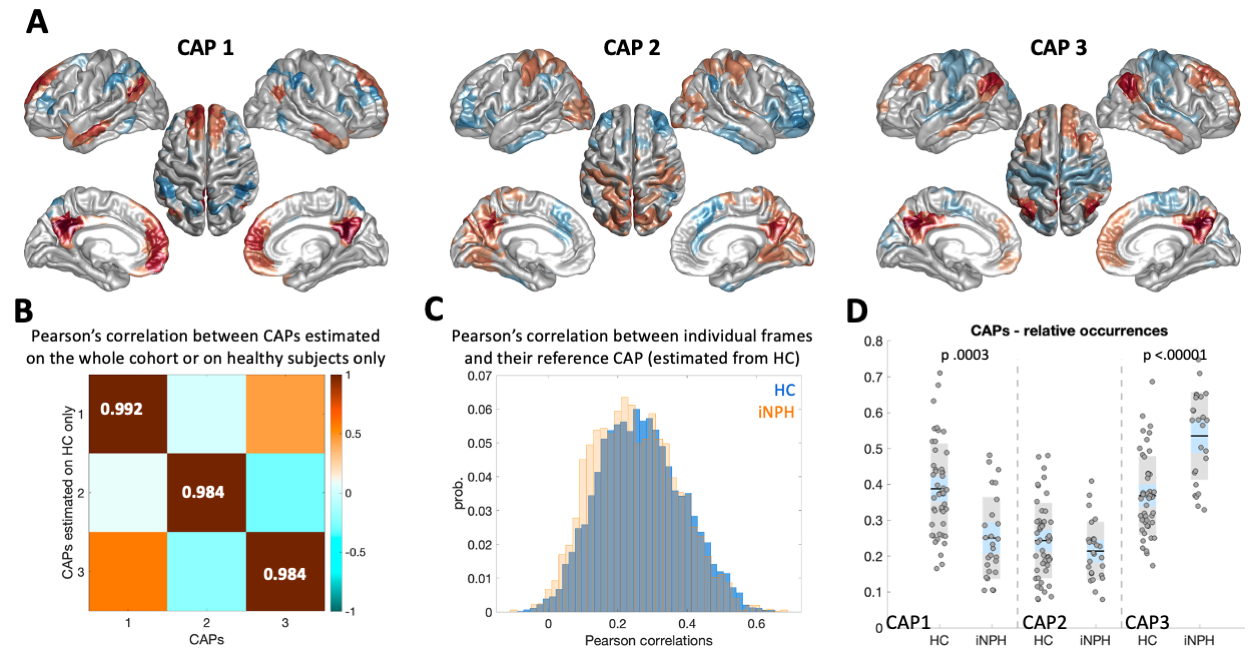


**Supplementary Figure 12.**

**Dynamic balance between CAPs’ occurrences.**

The occurrences of **DMN-CAP and ECN-CAP were strongly** negatively correlated across subjects (*r*(72) = -0.79, *p* < 10^-16^; iNPH: *r*(24) = -0.79, *p* < 10^-5^; HC: *r*(46) = -0.67, *p* < 10^-6^) indicating a within-subject balancing effect of CAPs dynamics. **The occurrences of DMN-CAP and VSM-CAP were negatively correlated in healthy controls and in the whole group of subjects (*r*(72) = -0.34, *p* = .0031; iNPH: *r*(24) = -0.33, *p* = .10; HC: *r*(46) = -0.54, *p* = .000067).** Blue diamonds represent healthy controls; orange diamonds represent iNPH patients.


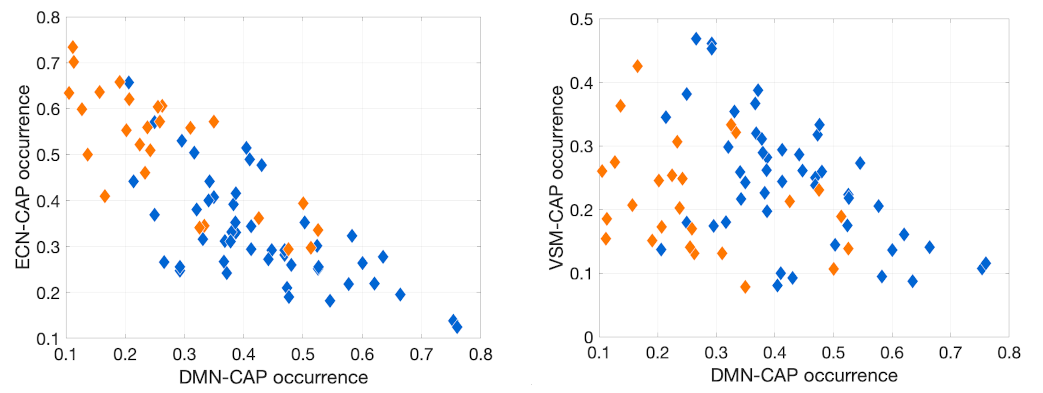


**Supplementary Figure 13.**

**CAPs represent pre- and post-CSF tap test data.**

Histograms of Pearson’s correlation values between individual fMRI frames corresponding to precuneus activations and the most similar CAP, for HCs and iNPH patients before the CSF tap test (grey bars) and for the iNPH patients after the CSF tap test. The good overlap between the two histograms indicates that the CAPs estimated on the HC and iNPH patients before the CSF tap test are spatially well representative of the precuneus dynamics occurring in iNPH patients after the CSF tap test.


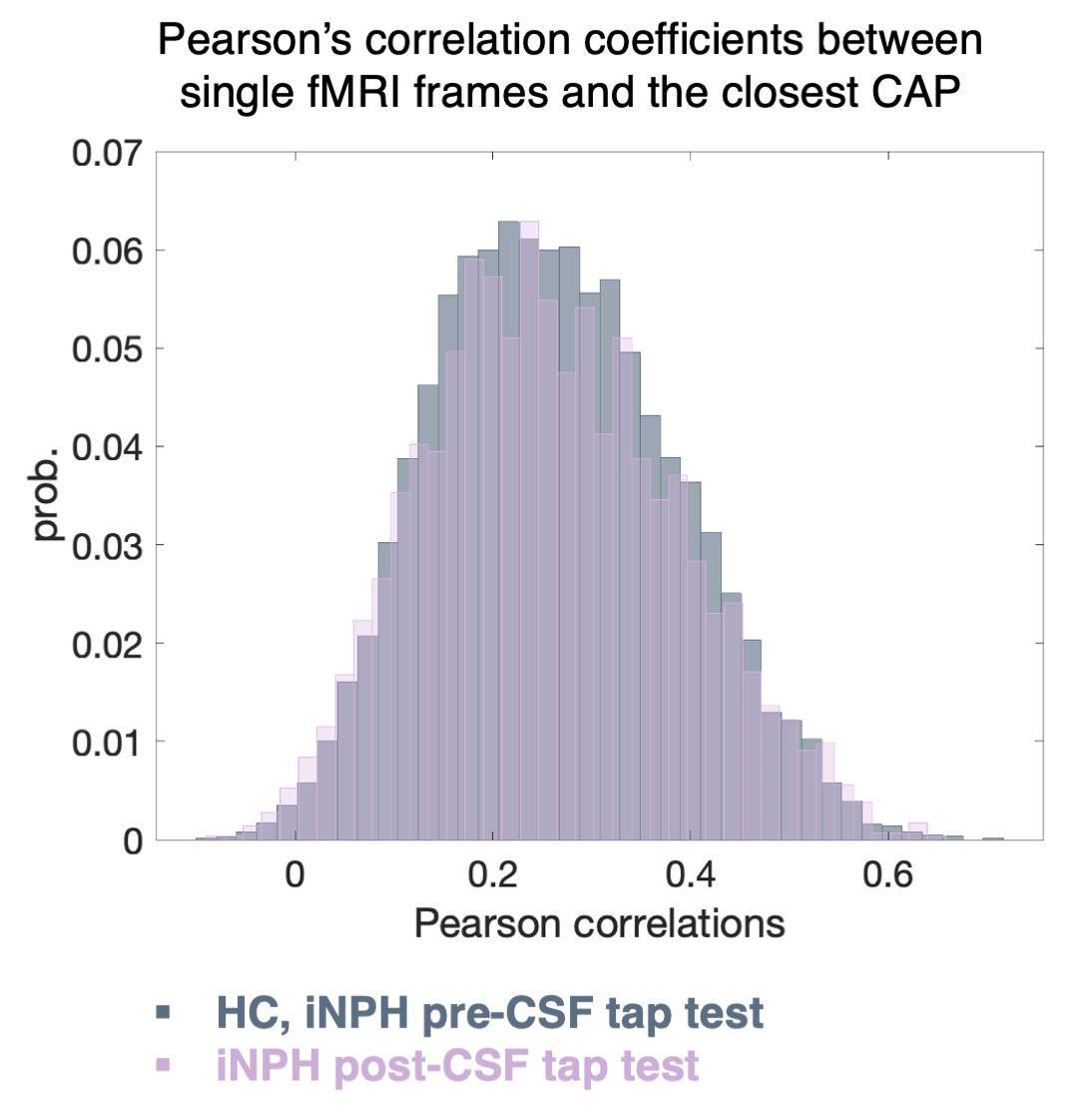


**Supplementary Figure 14.**

**Functional and gait changes after CSF tap test and AD biomarkers.**

**Scatterplots representing the percentage changes of brain function (CAP2_VSM_ and CAP3_ECN_ occurrences) and gait performances (walking speed and stride length) in iNPH patients after the CSF tap test, with respect to pre-CSF tap test value. Each diamond represents a single iNPH patient. The color-coding represents the patient’s status with respect to the AD biomarkers’ positivity/negativity.**


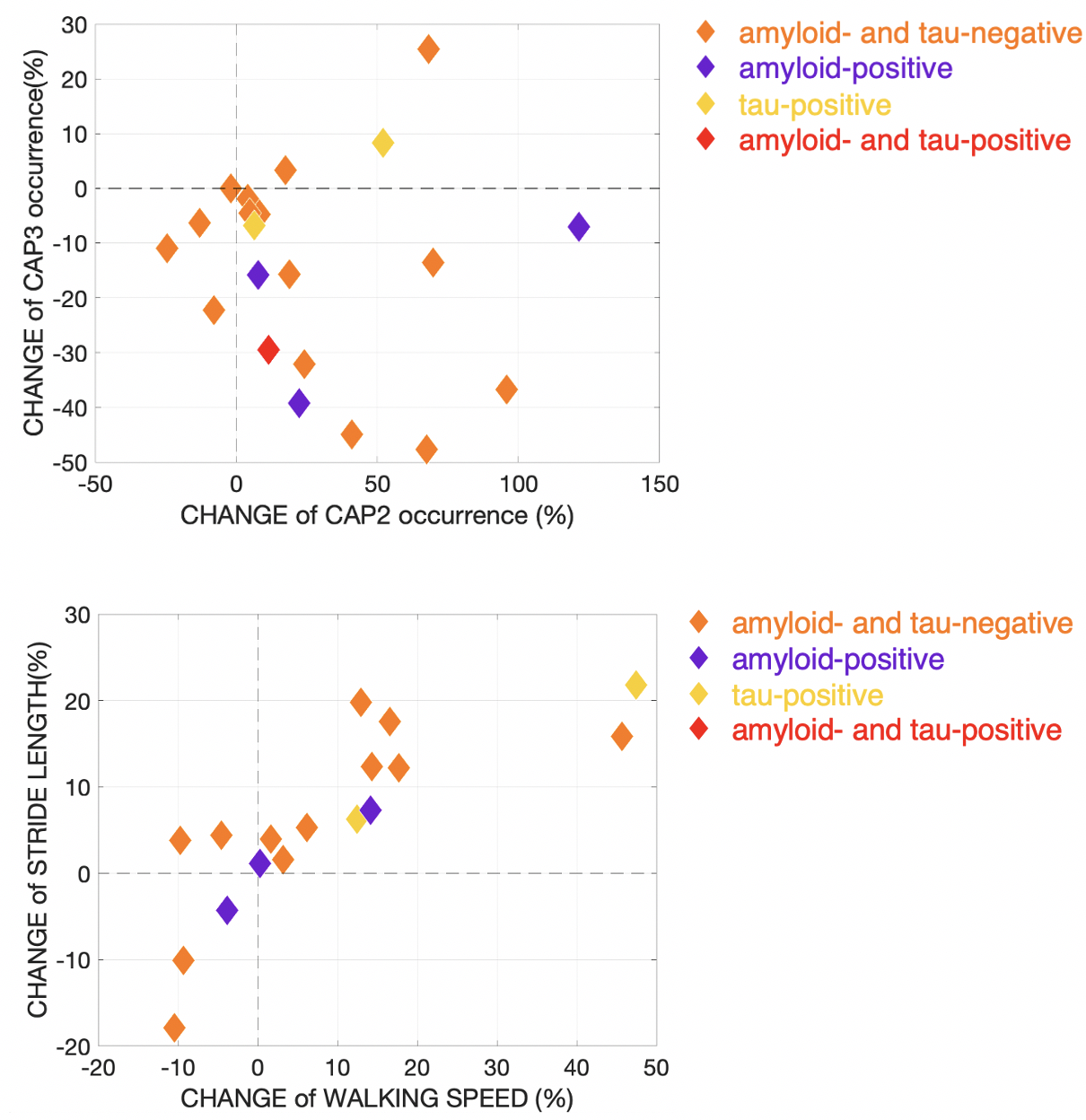


**Supplementary Figure 15.**

**iNPH-HC functional connectivity differences: comparison between preprocessing pipelines with and without global signal regression**

**Group-average functional connectivity matrices (first row) and effect sizes from iNPH-HC group-comparisons (second row) obtained from data WITHOUT global signal regression (left column) and WITH global signal regression (right column). When the global signal is regressed out from the fMRI time series, we observe an overall decrease of the functional connectivity values. The connectivity decrease involves in particular the interactions between lower-order and higher-order/default mode networks. The effect sizes for the iNPH-HC group-comparisons obtained from data with and without global signal regression are strongly correlated (bottom right scatterplot). However, the iNPH-HC p-values’ rankings obtained from data with and without global signal regression do not correspond closely (p-values < 0.05, < 0.01, and surviving multiple comparison correction are marked on the effect size matrices).**


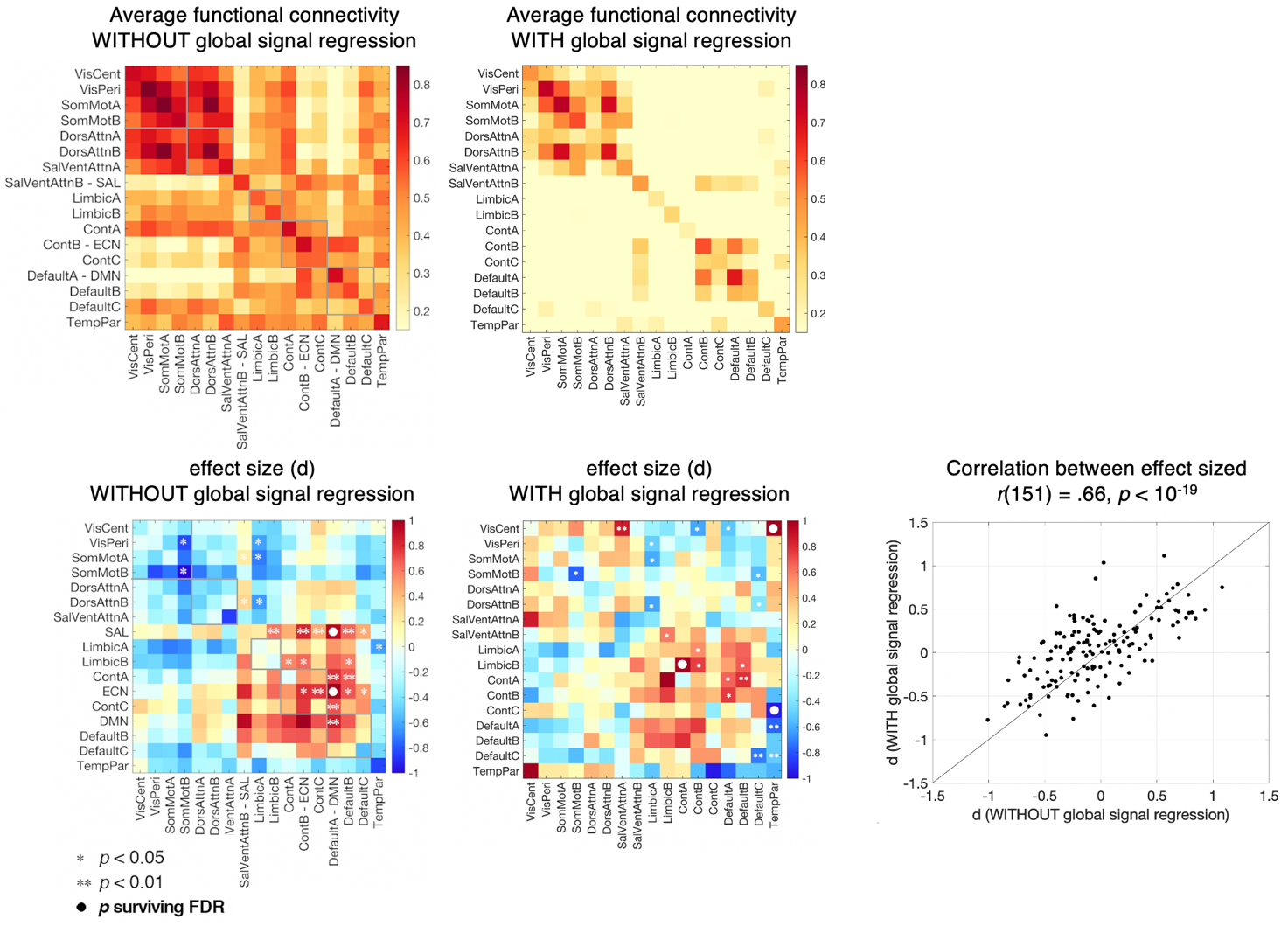


**Supplementary Figure 16.**

**Co-activation patterns (CAPs) are altered in iNPH and normalize after CSF tap test: Results’ replication from data with global signal regression**

**CAPs and subject-wise CAP’s statistics were derived from fMRI data with global signal regression. iNPH-HC group comparisons replicate main results obtained without global signal regression, although with inferior statistical power. A. Z-scored precuneus co-activation patterns (CAPs) projected onto a standardized cortical surface. Only the 15% largest positive contributions and the 15% smallest negative contributions are represented in color. B. Fraction of fMRI volumes corresponding to precuneus activations in individual subjects of the iNPH and HC groups. C. CAPs’ occurrences in individual subjects, iNPH and HC groups. D. CAPs’ occurrences in iNPH patients assessed before (‘pre’) and after (‘post’) the CSF tap test. Grey segments link the same subjects in the pre- and post-CSF tap test groups. P-values smaller than 0.1 are reported above the corresponding boxplots. Boxplots: black lines indicate group means; light blue areas represent 95% confidence intervals; grey areas represent 1 standard deviation intervals; raw data are jittered for better visualization.**


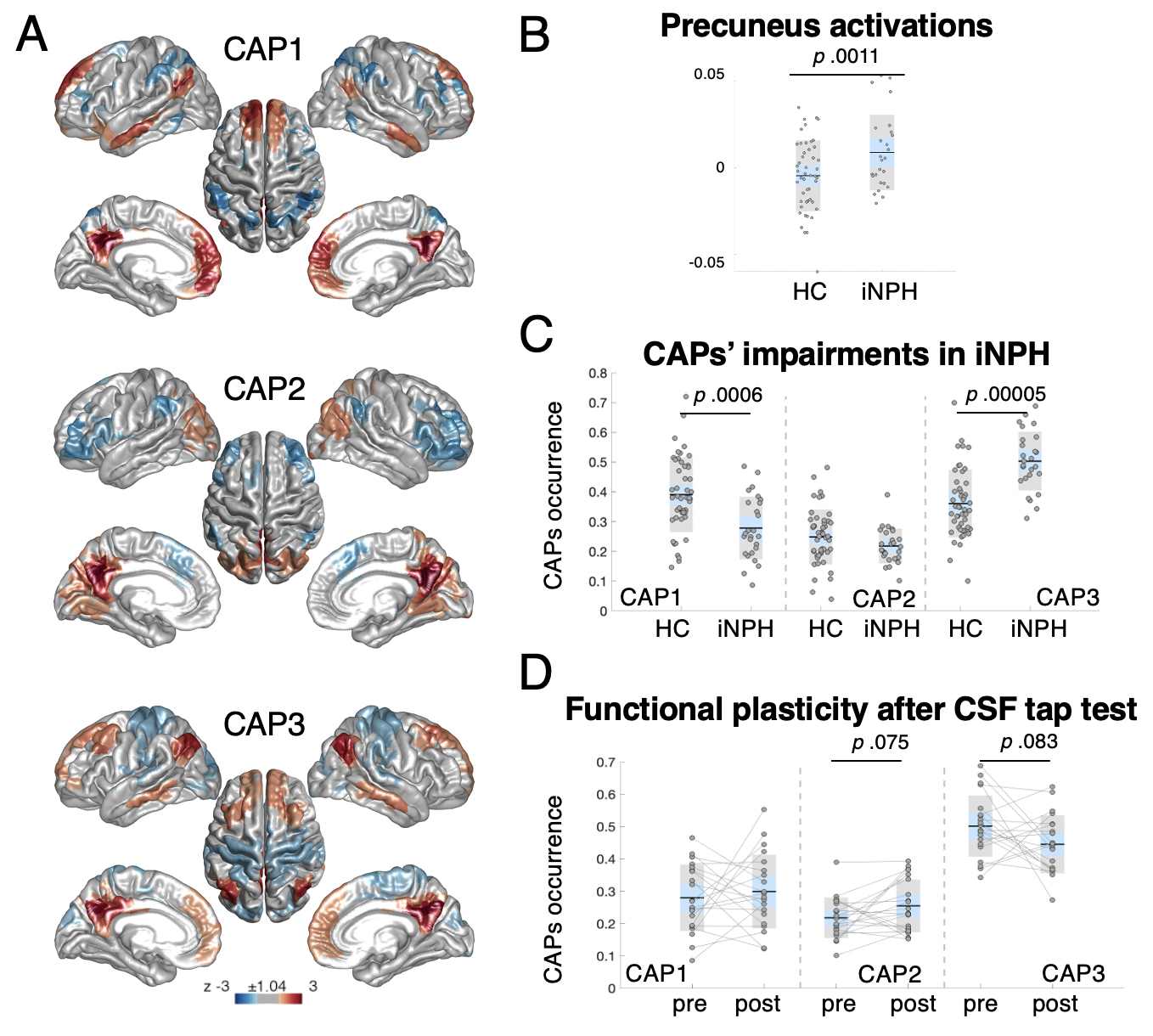


**Supplementary Figure 17.**

**Cortical clusters of increased co-activation with the precuneus: Results’ replication from data with global signal regression**

**Precuneus co-activation maps were obtained from fMRI data with global signals regression and compared between iNPH patients and healthy controls for the two contrasts iNPH<HC, iNPH>HC (permutation testing and threshold-free cluster enhancement using *FSL randomise*). No voxel had decreased co-activation with the precuneus in iNPH compared to HC. Conversely, 3 voxel clusters had increased co-activation with the precuneus (voxel-level corrected *p* < .05). The clusters are represented with distinct colors in the figure and correspond to the left dorsolateral prefrontal cortex, left intraparietal sulcus and left angular gyrus, in good agreement with main results.**


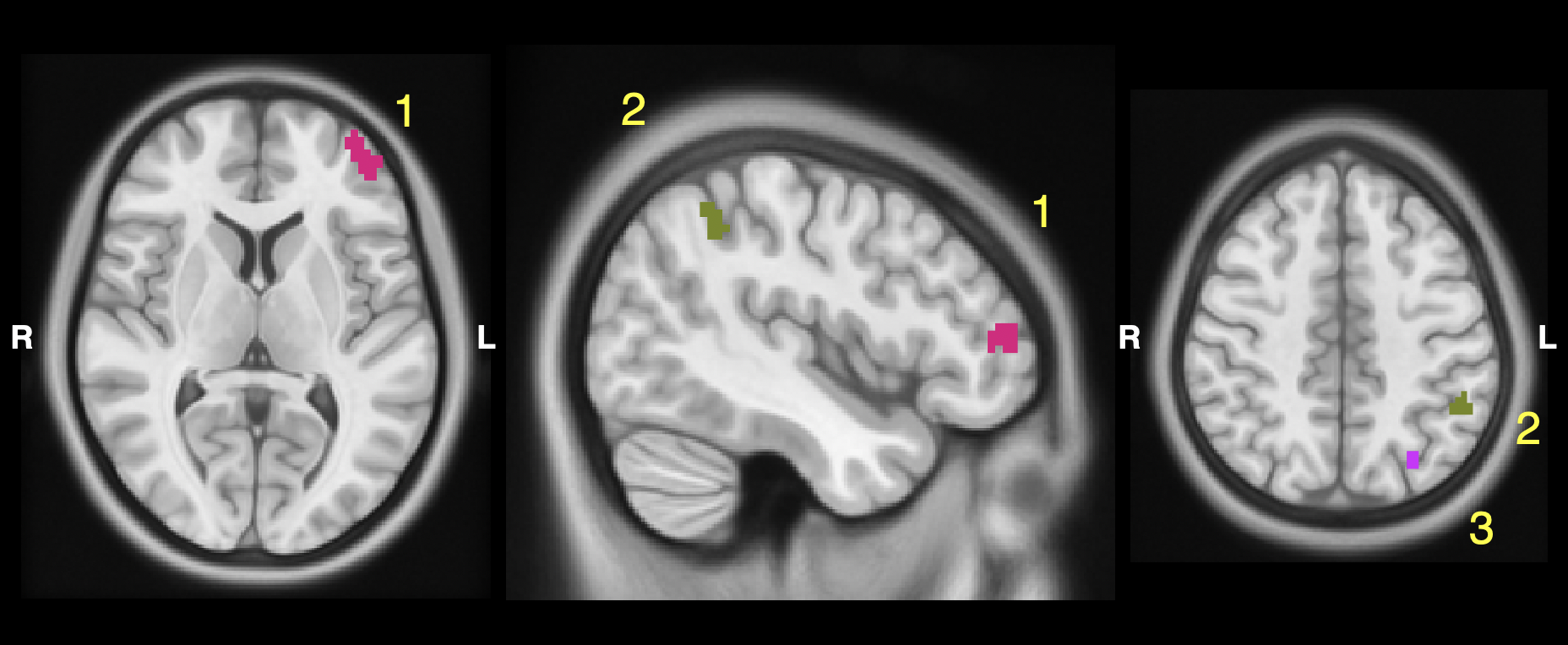


**References**

Bolton, T. A. W., Tuleasca, C., Rey, G., Wotruba, D., Gaviria, J., Dhanis, H., … Van De Ville, D. (2019). TbCAPs: A ToolBox for Co-Activation Pattern Analysis. *ArXiv:1910.06113 [q-Bio]*. Retrieved from http://arxiv.org/abs/1910.06113

Fonov, V., Evans, A., McKinstry, R., Almli, C., & Collins, D. (2009). Unbiased nonlinear average age-appropriate brain templates from birth to adulthood. *NeuroImage*, *47*, S102. https://doi.org/10.1016/S1053-8119(09)70884-5

Monti, S., Tamayo, P., Mesirov, J., & Golub, T. (2003). Consensus Clustering: A Resampling-Based Method for Class Discovery and Visualization of Gene Expression Microarray Data. *Machine Learning*, *52*(1), 91–118. https://doi.org/10.1023/A:1023949509487

Schaefer, A., Kong, R., Gordon, E. M., Laumann, T. O., Zuo, X.-N., Holmes, A. J., … Yeo, B. T. T. (2018). Local-Global Parcellation of the Human Cerebral Cortex from Intrinsic Functional Connectivity MRI. *Cerebral Cortex*, *28*(9), 3095–3114. https://doi.org/10.1093/cercor/bhx179

Yarkoni, T., Poldrack, R. A., Nichols, T. E., Van Essen, D. C., & Wager, T. D. (2011). Large-scale automated synthesis of human functional neuroimaging data. *Nature Methods*, *8*(8), 665–670. https://doi.org/10.1038/nmeth.1635

Yeo, B. T., Krienen, F. M., Sepulcre, J., Sabuncu, M. R., Lashkari, D., Hollinshead, M., … Buckner, R. L. (2011). The organization of the human cerebral cortex estimated by intrinsic functional connectivity. *Journal of Neurophysiology*, *106*(3), 1125–1165. https://doi.org/10.1152/jn.00338.2011
